# Impaired Magnesium Protoporphyrin IX Methyltransferase (ChlM) Impedes Chlorophyll Synthesis and Plant Growth in Rice

**DOI:** 10.3389/fpls.2017.01694

**Published:** 2017-09-28

**Authors:** Zhaohai Wang, Xiao Hong, Keke Hu, Ya Wang, Xiaoxin Wang, Shiyun Du, Yang Li, Dandan Hu, Kexin Cheng, Baoguang An, Yangsheng Li

**Affiliations:** ^1^State Key Laboratory of Hybrid Rice, Key Laboratory for Research and Utilization of Heterosis in Indica Rice, Ministry of Agriculture, The Yangtze River Valley Hybrid Rice Collaboration Innovation Center, College of Life Sciences, Wuhan University, Wuhan, China; ^2^The Key Laboratory of Crop Physiology, Ecology and Genetic Breeding, Ministry of Education, School of Agricultural Sciences, Jiangxi Agricultural University, Nanchang, China; ^3^Rice Research Institute, Anhui Academy of Agricultural Sciences, Hefei, China

**Keywords:** chlorophyll synthesis, light intensity, magnesium protoporphyrin IX methyltransferase (ChlM), photoperiod, plant growth, rice (*Oryza sativa*), *yellow-green leaf 18* (*ygl18*)

## Abstract

Magnesium protoporphyrin IX methyltransferase (ChlM) catalyzes the formation of magnesium protoporphyrin IX monomethylester (MgPME) from magnesium protoporphyrin IX (MgP) in the chlorophyll synthesis pathway. However, no *ChlM* gene has yet been identified and studied in monocotyledonous plants. In this study, a spontaneous mutant, *yellow-green leaf 18* (*ygl18*), was isolated from rice (*Oryza sativa*). This mutant showed yellow-green leaves, decreased chlorophyll level, and climate-dependent growth differences. Map-based cloning of this mutant identified the *YGL18* gene LOC_Os06g04150. *YGL18* is expressed in green tissues, especially in leaf organs, where it functions in chloroplasts. YGL18 showed an amino-acid sequence similarity to that of ChlM from different photosynthetic organisms. *In vitro* enzymatic assays demonstrated that YGL18 performed ChlM enzymatic activity, but ygl18 had nearly lost all ChlM activity. Correspondingly, the substrate MgP was largely accumulated while the product MgPME was reduced in *ygl18* leaves. *YGL18* is required for light-dependent and photoperiod-regulated chlorophyll synthesis. The retarded growth of *ygl18* mutant plants was caused by the high light intensity. Moreover, the higher light intensity and longer exposure in high light intensity even made the *ygl18* plants be more susceptible to death. Based on these results, it is suggested that *YGL18* plays essential roles in light-related chlorophyll synthesis and light intensity–involved plant growth.

## Introduction

Chlorophyll is the main component of the photosynthetic pigments found in plants, algae and cyanobacteria (Czarnecki and Grimm, 2012). Chlorophyll is extremely important in photosynthesis because of its essential roles in harvesting light energy to form chemical energy (Grossman et al., 1995; Fromme et al., 2003).

Chlorophyll synthesis from glutamyl-tRNA to chlorophyll *b* requires a 15-step of enzymatic reaction, and all 27 genes encoding these 15 enzymes have been identified in higher plants represented by Arabidopsis (*Arabidopsis thaliana*) (Beale, 2005; Nagata et al., 2005). However, only 10 genes encoding seven enzymes in the chlorophyll synthesis pathway have been identified in rice (*Oryza sativa*). Coproporphyrinogen III oxidase converts coproporphyrinogen III to protoporphyrinogen IX. *RLIN1*, encoding a putative coproporphyrinogen III oxidase in rice, was cloned from a lesion mimic mutant (Sun et al., 2011). Magnesium chelatase consists of three subunits (ChlH, ChlI, and ChlD) and it catalyzes the formation of Mg-protoporphyrin IX from protoporphyrin IX. *OsChlH* was identified via a T-DNA insertional mutant that had the chlorina and lethal phenotype (Jung et al., 2003). The *OsChlD* and *OsChlI* genes were cloned from the spontaneous chlorina mutants, *chl1* and *chl9*, respectively (Zhang et al., 2006). *OsChlD* was also cloned with the spontaneous yellow-green leaf mutant *ygl7*, and the RNAi plants of *OsChlD* were found to have a lethal phenotype (Deng et al., 2014). *OsCRD1* encodes Mg-Protoporphyrin IX monomethyl ester cyclase, transforming magnesium protoporphyrin IX monomethylester (MgPME) into divinyl protochlorophyllide. *OsCRD1* was cloned by using a pale-green leaf mutant *m167* and a yellow leaf mutant *yl-1*, and found to have a dual role in chlorophyll synthesis and photosynthesis capacity (Sheng et al., 2017; Wang et al., 2017). NADPH:protochlorophyllide oxidoreductase catalyzes the photoreduction of protochlorophyllide to chlorophyllide. *OsPORB*, encoding the NADPH:protochlorophyllide oxidoreductase B, was cloned by using the faded green leaf mutant *fgl*, and it was found essential for chlorophyll synthesis under high light conditions (Sakuraba et al., 2013). *OsDVR* encodes the divinyl reductase, which catalyzes the conversion of divinyl chlorophyll(ide) *a* to monovinyl chlorophyll(ide) *a*; *OsDVR* was cloned by using the spontaneous yellow-green leaf mutant *824ys* (Wang et al., 2010). Chlorophyll synthase catalyzes the esterification of chlorophyllide to form chlorophyll *a*, and *YGL1*, encoding the chlorophyll synthase, has been cloned with the chlorophyll-deficient mutant *ygl1* (Wu et al., 2007). Chlorophyll *b* is synthesized from chlorophyll *a* by chlorophyll *a* oxygenase; both *OsCAO1* and *OsCAO2* genes were identified to encode the chlorophyll *a* oxygenase; the *Tos17* insertion mutant of *OsCAO1* showed a pale green leaf phenotype, but the insertion mutant of *OsCAO2* was similar to the wild type (Lee et al., 2005). *OsCAO1* was also identified through the pale green leaf mutant *pgl*, impacting leaf senescence and thus indirectly affecting grain yield and quality (Yang et al., 2016).

Magnesium protoporphyrin IX methyltransferase (ChlM) is another key enzyme in the chlorophyll synthesis pathway. The *ChlM* gene has been identified and studied in several species. Through cDNA cloning and protein expression, the enzymatic kinetics of the ChlM in *Synechocystis* sp. PCC6803 have been elaborately studied: it catalyzes the methyl transfer from the common methyl donor *S*-adenosylmethionine (SAM) to the carboxyl group of the C13 propionate side chain of magnesium protoporphyrin IX (MgP), enabling the formation of MgPME and *S*-adenosylhomocysteine (Shepherd et al., 2003; Shepherd and Hunter, 2004). The structure of *Synechocystis* ChlM has also been analyzed to illustrate its catalytic mechanism (Chen et al., 2014). Two *Chlamydomonas reinhardtii* mutants defective in *ChlM* were identified; both were yellow in the dark and dim light, and their growth was inhibited under higher light intensities (Meinecke et al., 2010). In tobacco (*Nicotiana tabacum*), *ChlM* cDNA was cloned to study its enzymatic function, and ChlM was found to interact with the ChlH subunit of Mg chelatase (Alawady et al., 2005). Inhibited and excessive expression of tobacco *ChlM* revealed its functions involved in the inverse activation of protoheme and Mg porphyrin synthesis (Alawady and Grimm, 2005). In *Arabidopsis*, the *ChlM* gene was identified by cDNA cloning and the ChlM enzymatic activity was verified *in vitro* (Block et al., 2002). In addition, an *Arabidopsis* knock-out mutant of the *ChlM* gene showed that this gene is essential to form chlorophyll and subsequently for the formation of photosystem complexes (Pontier et al., 2007). Recently, it was proposed that ChlM was regulated by the NADPH-dependent thioredoxin reductase C on the redox status of conserved cysteine residues of ChlM (Richter et al., 2013). Further research has showed that three conserved cysteines are essential for the activity and redox regulation of *Arabidopsis* ChlM (Richter et al., 2016). A study of the pea plant (*Pisum sativum*) revealed that the ChlM activity is dependent on its folate status (Van Wilder et al., 2009). However, no *ChlM* gene has yet been identified and studied in monocotyledonous plants.

This study performed a map-based cloning of the *yellow-green leaf 18 (ygl18)* locus in rice. Doing so revealed that *ygl18* harbored a single-base substitution in the *OsChlM* gene, resulting in a single amino acid change in the protein. The ChlM activity of YGL18 was checked, while ygl18 was attenuated on the enzymatic function, leading to the accumulation of MgP and the reduction of MgPME in the *ygl18* mutant. On the basis of the phenotypic and molecular characterization of *ygl18, YGL18* is proposed to play multiple yet important roles in chlorophyll synthesis and plant growth.

## Materials and methods

### Plant materials and growth conditions

The *ygl18* mutant was isolated from the rice cultivar “Guangzhan63S” (*Oryza sativa* L. subsp. *indica*), and its yellow-green leaf phenotype was stably inherited. For our experiments, the plants were grown in the paddy field, green house, or plant growth chambers. For the experimental greening of the etiolated seedlings, wild-type and *ygl18* seeds were germinated and then grown in the chamber (30°C, 24 h dark) for 6 days, before exposure to light (150 μmol m^−2^ s^−1^). For expression patterns of *YGL18* in different tissues, wild-type and *ygl18* plants were grown in the chamber (150 μmol m^−2^ s^−1^ light/dark, 10/14 h, 26/22°C). For daily expression experiments, wild-type and *ygl18* seedlings were cultured in the chamber (28°C) for 12 days under short-day (8 h light, 150 μmol m^−2^ s^−1^) or long day (14 h light, 150 μmol m^−2^ s^−1^) conditions before sampling.

### Analysis of pigments

Chlorophyll was extracted from the plant leaves with ice-cold 80% acetone, and the chlorophyll contents per gram of leaf fresh weigh (FW) were determined as described previously (Lichtenthaler, 1987).

### Map-based cloning

To construct the genetic population, the rice cultivars “Nipponbare” (*Oryza sativa* L. subsp.*japonica*), “02428” (*Oryza sativa* L. subsp. *japonica*), and “9311” (*Oryza sativa* L. subsp.*indica*) were used in this study.

The F_2_ population from the cross between *ygl18* and “Nipponbare” were used for the preliminary mapping of the *YGL18* locus. Ninety-two F_2_ mutant plants were detected via simple sequence repeat markers, which were well distributed across all 12 chromosomes, thus allowing *ygl18* and “Nipponbare” to be distinguished. Five new markers (Y11, Y13, Y15, Y20, and Y37) were successfully developed to distinguish *ygl18* and “9311” based on the DNA sequence differences between the *indica* and *japonica* rice varieties. Therefore, the F_2_ population from the cross between *ygl18* and “9311” were used for the fine mapping, for which a total of 910 F_2_ mutant plants were tested. Primer sequences of the markers were listed in Table S1.

### Complementation of *YGL18*

To test the C-to-T mutation, the PCR amplification products were sequenced with the primer pair *YGL18* Mutation (Table S2). For complementation of the *ygl18* phenotype, two DNA fragments covering the 2,732-bp upstream promoter, the 981-bp gene region and the 1,433-bp downstream terminator of the gene LOC_Os06g04150, were firstly amplified from the genomic DNA of wild-type Guangzhan63S by using the primers *YGL18*C1 and *YGL18*C2 (Table S2). Then, the amplified products were constructed into the binary vector pBWA(V)HII (reconstructed from pCAMBIA1301), by using the *Aar* I restriction site on *YGL18*C1 and *YGL18*C2, and the *Bsa* I restriction site on pBWA(V)HII for the digestion-link reactions. The resultant seamlessly cloned 5,146-bp DNA fragment was sequenced to confirm its identity with the sequence found in the wild-type Guangzhan63S. This vector constructed for functional complementation was termed p*YGL18*C, while the empty pBWA(V)HII was renamed pEmvC. Both vectors p*YGL18*C and pEmvC were transformed into the calli of *ygl18* mutant through the *Agrobacterium* (EHA105)-mediated method. Positive transgenic plants were identified by using *HYG* primer pairs (Table S2) specific for the amplification of the *hygromycin* gene.

### Bioinformatic analysis

To reveal the specific ChlM encoded by *YGL18*, BLASTP searches were conducted with the full-length amino acid sequence on NCBI (http://www.ncbi.nlm.nih.gov/). The *YGL18* gene information was further investigated on Rice Genome Annotation Project website (http://rice.plantbiology.msu.edu/). Full-length amino acid sequences were used for multiple sequence alignments performed with the ClustalX2 software. A consensus phylogenetic tree was built with the MEGA5 software by using the neighbor-joining method with 1,000 random bootstrap replicates.

The online software ChloroP 1.1 Server (http://www.cbs.dtu.dk/services/ChloroP/) and TargetP 1.1 Server (http://www.cbs.dtu.dk/services/TargetP/) were used to predict the subcellular localization of YGL18.

### Gene expression analysis

The collected samples were immediately frozen in liquid nitrogen, and then stored at −80°C before use. Total RNAs of the various tissues were isolated with the TRIzol reagent (Invitrogen). After the DNase treatment, about 5 μg of RNAs were used for the cDNAs synthesis that utilized the M-MLV reverse transcriptase (Promega) in a 50-μl reaction mixture. Quantitative real-time reverse transcription polymerase chain reaction (qRT-PCR) was performed with the 2 × SYBR Green Master Mix reagent (Bio-Rad) in a 96-well plate of the Bio-Rad CFX96 real time PCR system. Eight rice reference genes: TI (LOC_Os01g05490), ARF (LOC_Os05g41060), EF-1α (LOC_Os03g08020), UBC (LOC_Os02g42314), Profilin-2 (LOC_Os06g05880), Edf (LOC_Os08g27850), PtfS (LOC_Os07g34589), and Actin1 (LOC_Os03g50885), were used to find the stable internal standards by geNorm as described (Vandesompele et al., 2002; Wang et al., 2016). The selected internal standards for the different experimental conditions were showed in Figure S1. All primers used for qRT-PCR analysis are listed in Table S3, with good PCR efficiencies (80–100%) detected by using ten-times diluted gradients of the total cDNAs.

### Subcellular localization of YGL18

To fuse YGL18 with the yellow fluorescent protein (YFP), the coding sequence (CDS) of *YGL18* without the termination codon was amplified by using the primer pair *YGL18*-*YFP* (*Bbs* I restriction site) (Table S2), and then cloned into the vector pBWD(LB)-p35SYFP (*Bsa* I restriction site). The fusion constructs (p35S::*YGL18*-*YFP*) and the control (p35S::*YFP*) were then transformed into the rice protoplasts as previously described (Yu et al., 2014). The samples were subsequently examined with an FV1000 confocal system (OLYMPUS FLUOVIEW).

### Recombinant protein construction, expression and purification

To generate the glutathione-*S*-transferase (GST) fusion constructs, *GST*-*YGL18* and *GST*-*ygl18*, 981-bp full-length coding sequences of *YGL18* and *ygl18* were amplified, respectively, by using the primer pair GST-*YGL18*/*ygl18* (Table S2), and then inserted into pGEX-6P-1 by using the restriction enzyme sites *Bam*H I and *Eco*R I. The subsequent recombinant protein construction, expression and purification were performed as described previously (Wang et al., 2015).

### ChlM enzymatic assay

The enzymatic reactions were performed as described previously (Richter et al., 2013) with some modifications. The reaction mixture included 50 mM Tris-HCl (pH7.5), 500 μM NADPH, 500 μM SAM, and 2 μM MgP in a final volume of 100 μl. After the addition of the recombinant proteins (GST, GST-YGL18, or GST-ygl18), the reactions were incubated at 30°C for 1 h. These reactions were stopped by adding 500 μl of acetone:NH_4_OH (9:1), and the product formation was determined by HPLC as described below.

### Extraction of MgP and MgPME from plant leaves

Plant leaves were sampled and frozen in liquid nitrogen, and then stored at −80°C before use. The harvested leaves (100 mg) were powdered in liquid nitrogen with a pestle and mortal. The MgP and MgPME contents were extracted by using chilled acetone according to the methods as previously described (Mochizuki et al., 2008).

### Quantification of MgP and MgPME by HPLC

The HPLC analysis of MgP and MgPME was conducted as previously described (Mochizuki et al., 2008) with some modifications. Briefly, here we used a ZORBAX SB-C18 column (5 μm, 4.6 mm × 250 mm), eluting with a linear gradient of solvent B (85% methanol, 15% 0.1 M ammonium acetate, pH5.2) in solvent A (55% H_2_O, 30% methanol, 15% 0.1 M ammonium acetate, pH5.2) as follows: 0 to 100% solvent B over 7 min and 100% solvent B for 16 min, with a flow rate of 1 mL min^−1^. To identify MgP and MgPME in samples, standard curves were constructed with the authentic standards for each independent experiment, as shown in Figure S2 (for *in vitro* ChlM enzymatic experiment) and Figure S3 (for content measurement in leaves). MgP was purchased from Frontier Science, and MgPME was chemically synthesized by treating MgP with diazomethane in methanol as previously described (Strand et al., 2003).

## Results

### The phenotype of *ygl18*

**Figure 1:**
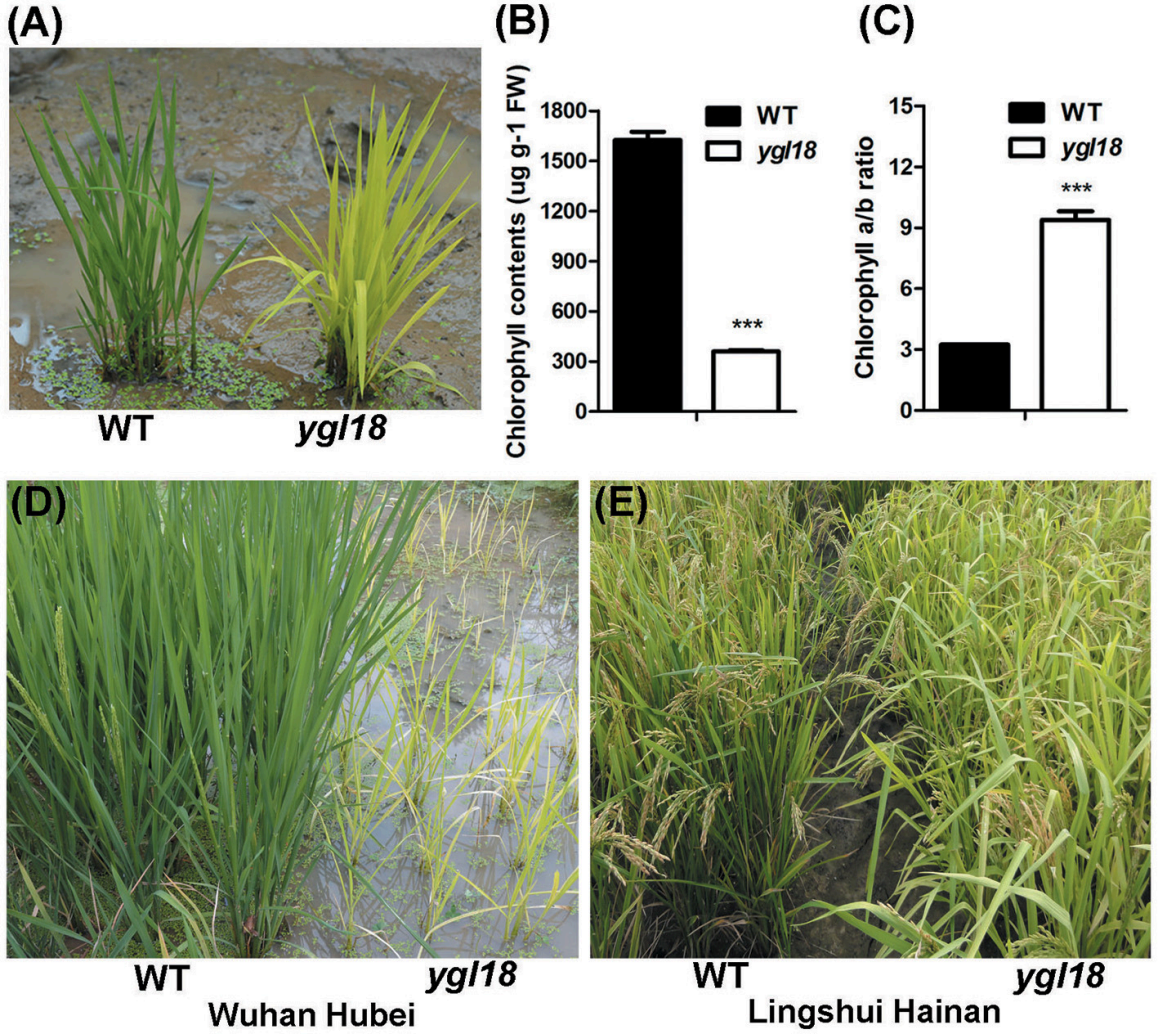
Phenotypic characterization of the wild-type and *ygl18* mutant plants. **(A)** Plants at the seedling stage (about 15 days old) in the paddy field. **(B)** Chlorophyll contents. FW, fresh weight. **(C)** Chlorophyll *a*/*b* ratio. **(B,C)** Data represent the mean ± SD of three biological replicates (Student's *t*-test: ^***^P < 0.0005). **(D)** Plants growing in a natural field in Wuhan of Hubei province in China. **(E)** Plants growing in a natural field in Lingshui of Hainan province in China.

The spontaneous *ygl18* mutant was isolated from the rice cultivar “Guangzhan63S” (*Oryza sativa* subsp. *indica*) during its reproduction. In the paddy field, the *ygl18* mutant plants could be easily distinguished by their yellow-green leaves at the seedling stage (Figure 1A). Upon examination, the chlorophyll content was greatly decreased in *ygl18* leaves when compared with that of the wild type (Figure 1B). But the chlorophyll *a*/*b* ratio appeared higher in the *ygl18* mutant (Figure 1C). When the plants were cultivated in a natural field in Wuhan (113°41′E-115°05′E, 29°58′N-31°22′N; Hubei province) from June to September, the *ygl18* mutants showed severely retarded growth (Figure 1D); sometimes the *ygl18* mutants could not even grow and just withered away. However, the growth status of the *ygl18* mutants was nearly similar to that of the wild type (Figure 1E), when the plants were cultivated in a natural field in Lingshui (109°45′E-110°08′E, 18°22′N-18°47′N; Hainan province) from December to March. Therefore, the *ygl18* phenotypic characterization implied that the *YGL18* is probably involved in chlorophyll synthesis and climate-dependent plant growth in rice.

### Map-based cloning of *YGL18*

For the genetic analysis, four hybrid populations were constructed from the crosses between the *ygl18* mutant and “Nipponbare,” “02428,” and “9311.” All F_1_ plants from these crosses displayed the wild-type green leaves, while their F_2_ progenies all showed a segregation ratio of 3:1 (green: yellow-green plants, χ^2^ < $\chi_{0.05}^{2}$ = 3.84; *P* > 0.05; Table S4). These results suggested that the yellow-green leaf phenotype of the *ygl18* mutant is controlled by a single recessive nuclear gene.

**Figure 2:**
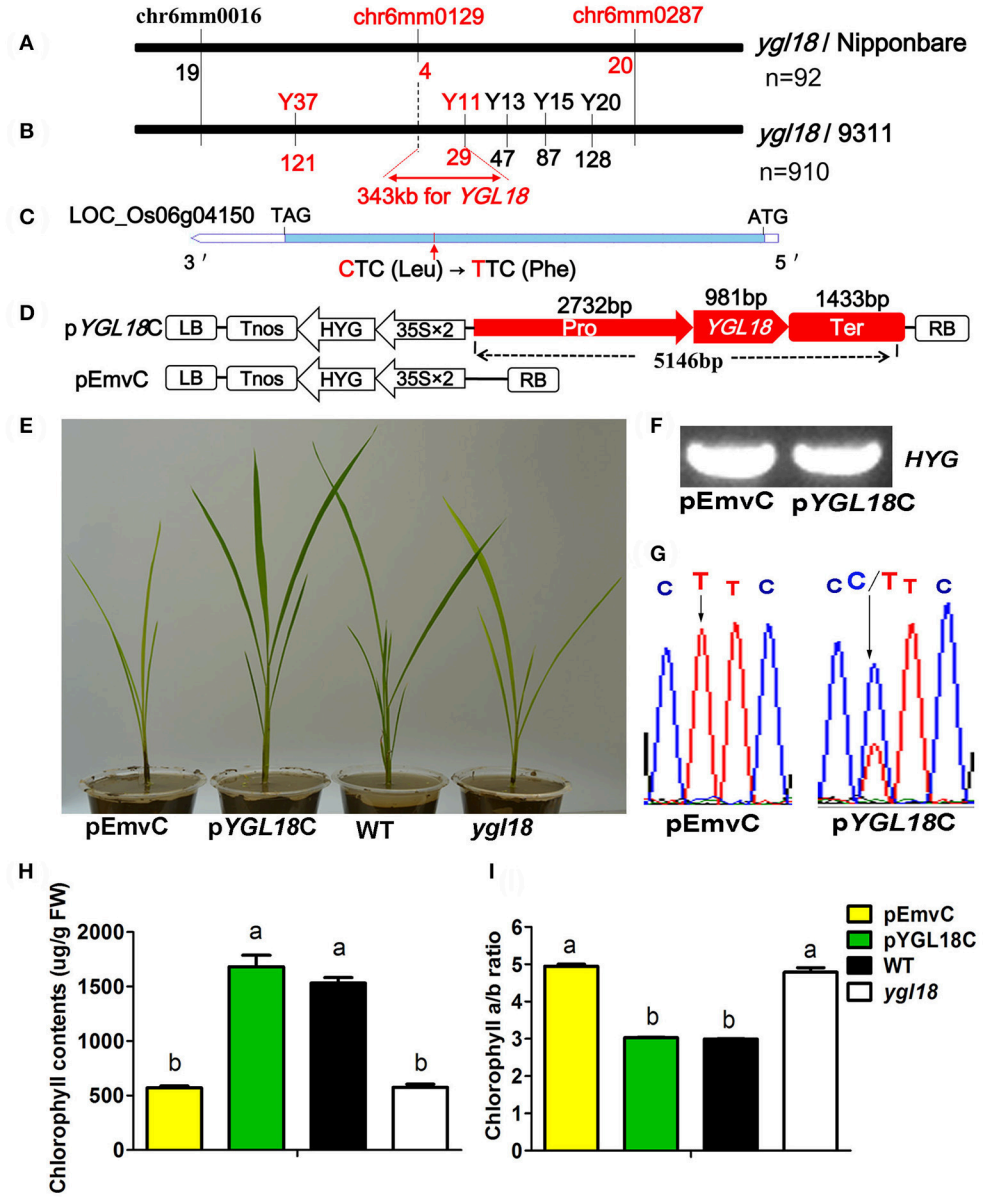
Map-based cloning and functional complementation of *YGL18*. **(A)** Preliminary mapping identified the *YGL18* locus as occurring between chr6mm0129 and chr6mm0287 on the rice chromosome 6, by using 92 F_2_ recessive individuals from the cross of *ygl18* and “Nipponbare.” The number of recombinants is indicated below the marker. **(B)** The *YGL18* locus was mapped to lie between markers Y37 and Y11 by 910 F_2_ recessive individuals from the cross of *ygl18* and “9311.” Accordingly, a candidate region of 343 kb between chr6mm0129 and Y11 was summarized. **(C)** Gene structure of the *YGL18* candidate, LOC_Os06g04150. The blue bar indicates the CDS region. The red arrow indicates the point mutation of C–T, leading to the amino acid exchange of Leu to Phe. **(D)** Vectors for the complementary experiment. The functional complementary vector p*YGL18*C contains the promoter (Pro), gene region (*YGL18*), and terminator (Ter) of LOC_Os06g04150; pEmvC is the empty vector control. LB, left border; Tnos, the *nopaline synthase* terminator; HYG, the *hygromycin* gene; 35S, cauliflower mosaic virus 35S promoter; RB, right border. **(E)** Transgenic plants of pEmvC and p*YGL18*C grown in the green house. **(F)** Positive amplification of the transgenic marker element (*HYG* gene) in the transgenic plants of pEmvC and p*YGL18*C. **(G)** Sequence analysis of the C-to-T mutation site in T_0_ transgenic plants of pEmvC and p*YGL18*C. **(H)** Chlorophyll contents in the transgenic plant leaves of pEmvC and p*YGL18*C. FW, fresh weight. **(I)** Chlorophyll *a*/*b* ratio in the transgenic plant leaves of pEmvC and p*YGL18*C. In **(H,I)** Data represent the mean ± SD of three biological replicates (one-way ANOVA analysis). Different letters indicate values are statistically different based on a one-way ANOVA analysis.

Ninety-two yellow-green plants, coming from the F_2_ cross population between the *ygl18* mutant and “Nipponbare,” were used for the preliminary mapping of the *YGL18* locus. The *YGL18* locus was mapped between the SSR makers chr6mm0129 and chr6mm0287 on chromosome 6 (Figure 2A), and no polymorphism marker between *ygl18* and “Nipponbare” was found in this region. However, five new markers (Y11, Y13, Y15, Y20, and Y37) in or beside the preliminary region were developed, showing polymorphism between *ygl18* and “9311.” Accordingly, by using 910 F_2_ yellow-green plants generated from the cross of *ygl18* and “9311,” the *YGL18* locus was additionally mapped between the new makers Y37 and Y11 (Figure 2B). As a result, the *YGL18* gene was eventually limited in a 343-kb region between markers chr6mm0129 and Y11 (Figure 2B). A total of 41 putative genes were predicted according to the NCBI website. Genomic sequences of the *ygl18* mutant were sequenced for all these 41 genes, and one point mutation was found on the CDS region of the 21st gene, LOC_Os06g04150 (Figure 2C). The single nucleotide substitution of cytosine (C) to thymine (T) occurred at the 673-bp position of the CDS, resulting in a single amino acid change from leucine (Leu) to phenylalanine (Phe). This C-to-T mutation was existed for *ygl18* only. It did not occur in nine different normal rice cultivars, and similarly, all the tested F_2_ yellow-green plants had T at this nucleotide site, while the tested F_2_ green plants owned the C or C/T (Table S5). These results suggested that LOC_Os06g04150 is the likely *YGL18* candidate.

A functional complementation experiment was conducted to confirm that the mutation in LOC_Os06g04150 was responsible for the *ygl18* phenotype. A 5146-bp genomic fragment of LOC_Os06g04150 from the wild-type Guangzhan63S was constructed into the plasmid p*YGL18*C and then transferred into the *ygl18* calli via an *Agrobacterium tumefaciens*-mediated transformation, while the empty vector pEmvC was transformed as the control (Figure 2D). The yellow-green leaves were completely restored to normal color in nine independent transgenic lines with p*YGL18*C, but they were unchanged in eight independent control transgenic lines with pEmvC (Figures 2E–G). The chlorophyll contents and the chlorophyll *a*/*b* ratio in the complementation lines with p*YGL18*C were also restored to normal, as they occur in the wild type (Figures 2H,I). Hence, these results confirmed that LOC_Os06g04150 indeed is the *YGL18* gene.

### *YGL18* is mainly expressed in leaves and localized in chloroplasts

**Figure 3:**
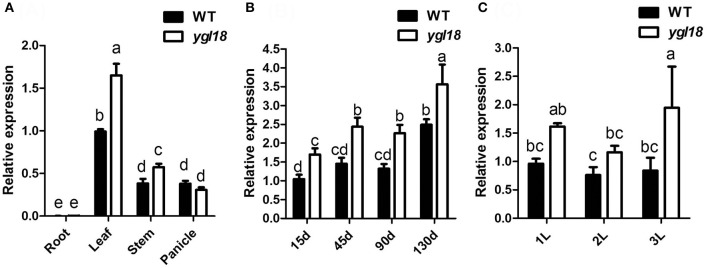
Expression analysis of *YGL18* by qRT-PCR. **(A)** Expression patterns of *YGL18* in root, leaf, stem and panicle of the wild type and *ygl18* mutant. Root and leaf were sampled from 15-day-old plants, while stem and panicle were sampled from 130-day-old plants. **(B)** Expression patterns of *YGL18* in the newest fully expanded leaves of wild-type and *ygl18* mutant plants at different days after germination. **(C)** Expression patterns of *YGL18* in the first, second, and third fully expanded leaves of the wild-type and *ygl18* mutant plants at about 90 days after germination. Expression levels of *YGL18* in the leaf of wild type **(A)**, the 15-day-old leaf of wild type **(B)**, and the first leaf of wild type **(C)**, were respectively normalized to 1. Data represent the mean ± SD of three or four biological replicates (one-way ANOVA analysis). Different letters indicate values are statistically different based on a one-way ANOVA analysis.

The expression patterns of *YGL18* in different rice tissues were analyzed by qRT-PCR. The *YGL18* mRNAs were most abundant in the leaf, moderate in the stem and panicle, with very few occurring in the root (Figure 3A). We further investigated the expression of *YGL18* in the top leaf of plants at different developmental time points. Expression levels of *YGL18* steadily increased in the leaves of 15-, 45-, 90-, and 130- day-old wild-type plants, and these levels were even higher in the leaves of *ygl18* plants compared with those of the wild type (Figure 3B). Expression levels of *YGL18* were also tested in the first, second, and third plant leaves from the top. While they were approximately equal among wild-type leaves, they were, respectively higher in the *ygl18* leaves when compared with those of the wild type (Figure 3C). These results suggested that *YGL18* mainly operates in the green tissues, especially in leaf organs, and that *YGL18* is upregulated in the *ygl18* mutant leaves.

**Figure 4:**
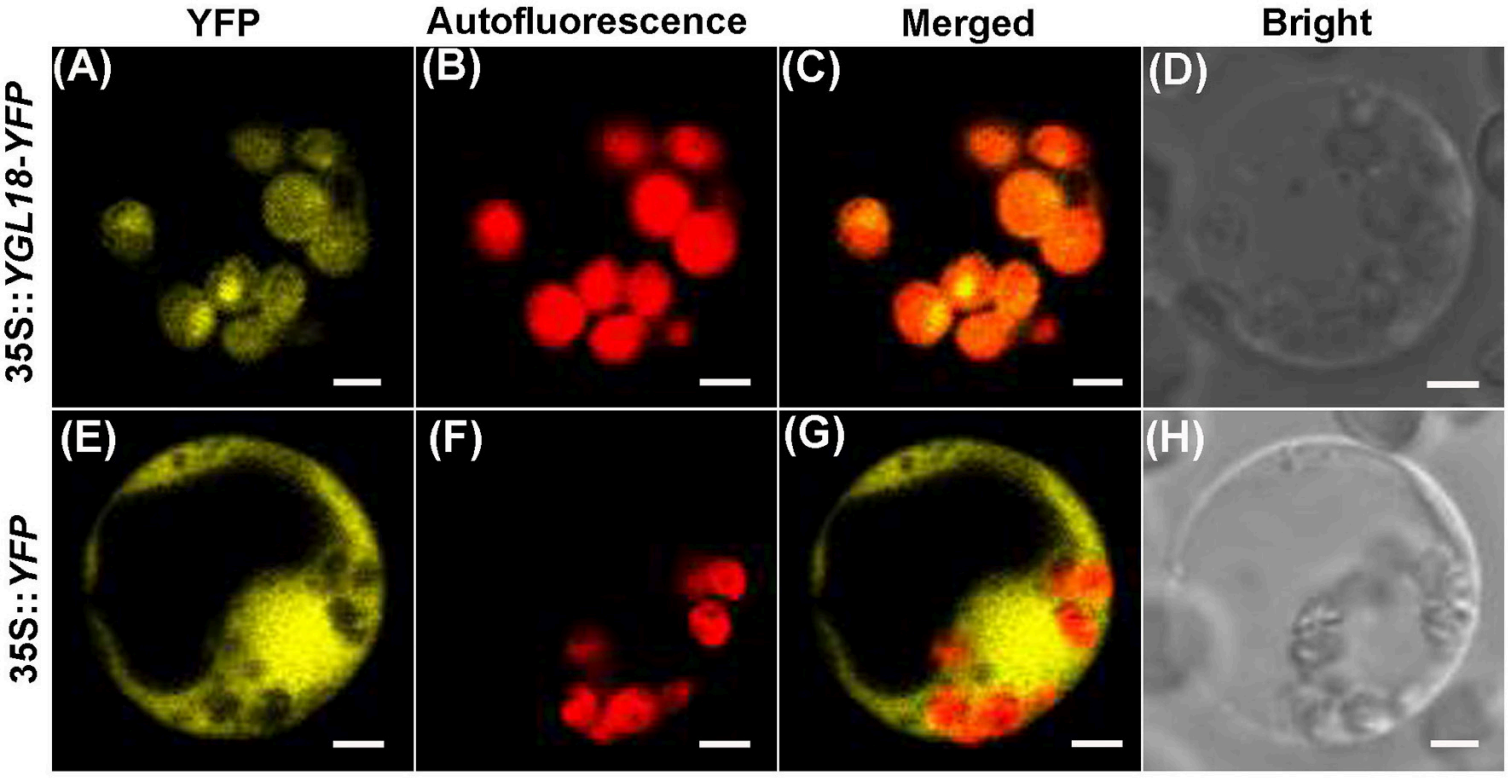
Subcellular localization of YGL18 in rice. Rice protoplast transformed with: **(A–D)** 35S::*YGL18*-*YFP*, and **(E–H)** the empty vector 35S::*YFP* as control. **(A,E)** YFP fluorescence images, **(B,F)** Chloroplast autofluorescence images, **(C,G)** Merged images of YFP fluorescence and chloroplast autofluorescence, **(D,H)** Bright field. Bar: 2 μm.

To reveal where the YGL18 protein functions in the cells, the online software ChloroP 1.1 Server and TargetP 1.1 Server were firstly used to predict the subcellular localization of YGL18. The YGL18 protein was found to contain chloroplast transit peptides and so it should target into the chloroplasts. To confirm this prediction, YGL18 was fused with YFP. The 35S::*YGL18*-*YFP* and 35S::*YFP* were separately introduced into the rice protoplasts. The YFP fluorescence signal was found to co-localize with the area of chloroplast autofluorescence in those protoplasts transformed with 35S::*YGL18*-*YFP* (Figures 4A–D). By contrast, the YFP fluorescence signal appeared throughout the cytoplasm, but not in the chloroplasts, when protoplasts were transformed with 35S::*YFP* (Figures 4E–H). These results indicated that the YGL18 protein is localized in the chloroplasts where it presumably performs its function.

### *YGL18* encodes a putative ChlM, and a conserved amino acid is substituted in the ygl18 protein

**Figure 5:**
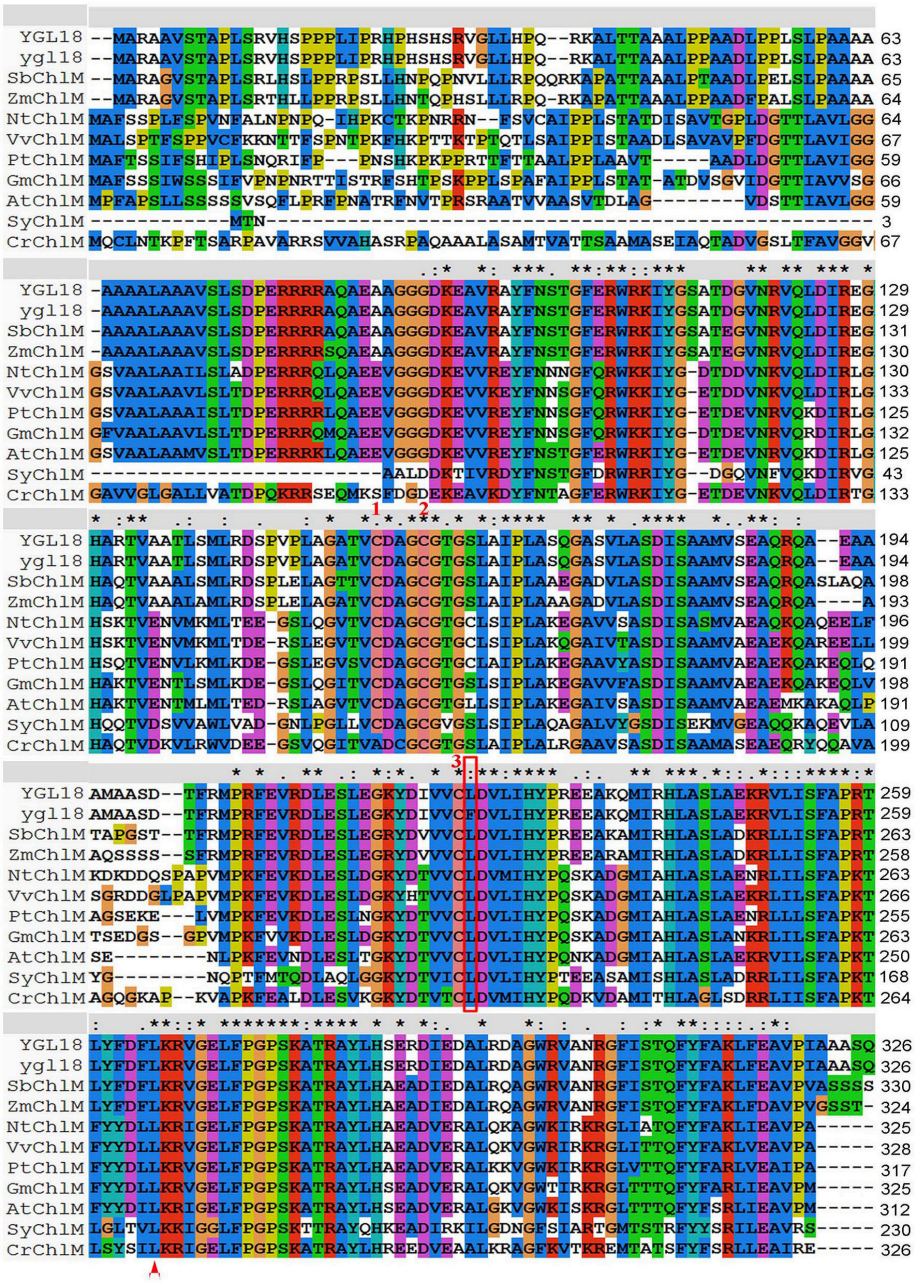
Multiple sequences alignment of YGL18 and ygl18 with their homologs in different photosynthetic organisms. The amino acid substitution of Leu (L) to Phe (F) at position 225 in ygl18 protein sequences is labeled in a red rectangular box. The Leu (L) at position 270 in CrChlM, which is substituted in *chlM-1* in *Chlamydomonas reinhardtii* (Meinecke et al., 2010), is indicated by the red triangle at the bottom. Three conserved cysteine residues for the redox regulation of ChlM activity (Richter et al., 2016), are indicated by the red number 1, 2, and 3, respectively, at the top. “^*^” fully conserved residue; “:” conservation between groups with strongly similar properties; “.” conservation between groups with weakly similar properties; “-“ gap. VvChlM, *Vitis vinifera* (XP_002280872.1); NtChlM, *Nicotiana tabacum* (AAF70243.1); PtChlM, *Populus trichocarpa* (XP_002318168.2); GmChlM, *Glycine max* (XP_003532350.3); AtChlM, *Arabidopsis thaliana* (NP_194238.1); OsChlM (YGL18), *Oryza sativa* (XP_015641356.1); SbChlM, *Sorghum bicolor* (XP_002436414.1); ZmChlM, *Zea mays* (AFW75394.1); CrChlM, *Chlamydomonas reinhardtii* (EDO97000.1); SyChlM, *Synechocystis sp. PCC 6803* (BAA10812.1).

There is only one copy of the *YGL18* gene in the rice genome as detected by NCBI blastn search. Specifically, the 981-bp CDS encoding a protein of 326 amino acids is predicted on Rice Genome Annotation Project website. The NCBI blastp revealed that *YGL18* is the *ChlM* gene, encoding a putative ChlM. Alignment of the YGL18 and ygl18 proteins with their homologs from nine different photosynthetic organisms was also performed. The result showed that many of the amino acid residues—in the region from 95 to 319 in the YGL18 amino acid sequences—were highly conserved among the tested organisms, and the amino acid substitution of Leu (L) to Phe (F) at position 225 in the ygl18 amino acid sequences was originally a fully conserved residue (Figure 5), implying the Leu at this position was biologically important. We then analyzed the possible phylogenetic relationships between YGL18 and its homologs by constructing a bootstrap consensus phylogenetic tree. The rice YGL18 was more closely related to the ChlM orthologs of monocotyledonous plants *Sorghum bicolor* and *Zea mays* than to those of other species (Figure S4). In addition, the YGL18 protein shared over 50% amino acid identity with all the analyzed ChlM orthologues in different photosynthetic organisms, including the prokaryote *Synechocystis* sp. PCC6803 (Figure S4). Together, these results suggested that *YGL18* encodes a putative and functionally conserved ChlM protein in rice, and that the substitution of the single conserved amino acid in ygl18 protein may impact its function.

### YGl18 performs ChlM enzymatic activity while the function of ygl18 is starkly weakened

To test the ChlM activity of the YGL18 and ygl18 proteins, recombinant GST-YGL18 and GST-ygl18 proteins were produced. Theoretically, the molecular weights of GST, YGL18, and ygl18 proteins are 26, 34.93, and 34.96 kDa, respectively. After induction, GST, GST-YGL18 (about 61 kDa), and GST-ygl18 (about 61 kDa) were highly expressed at the expected sizes (Figure S5, lanes 2–4). Column-purified proteins were used to conduct the enzymatic reactions (Figure S5, lanes 5–7).

**Figure 6:**
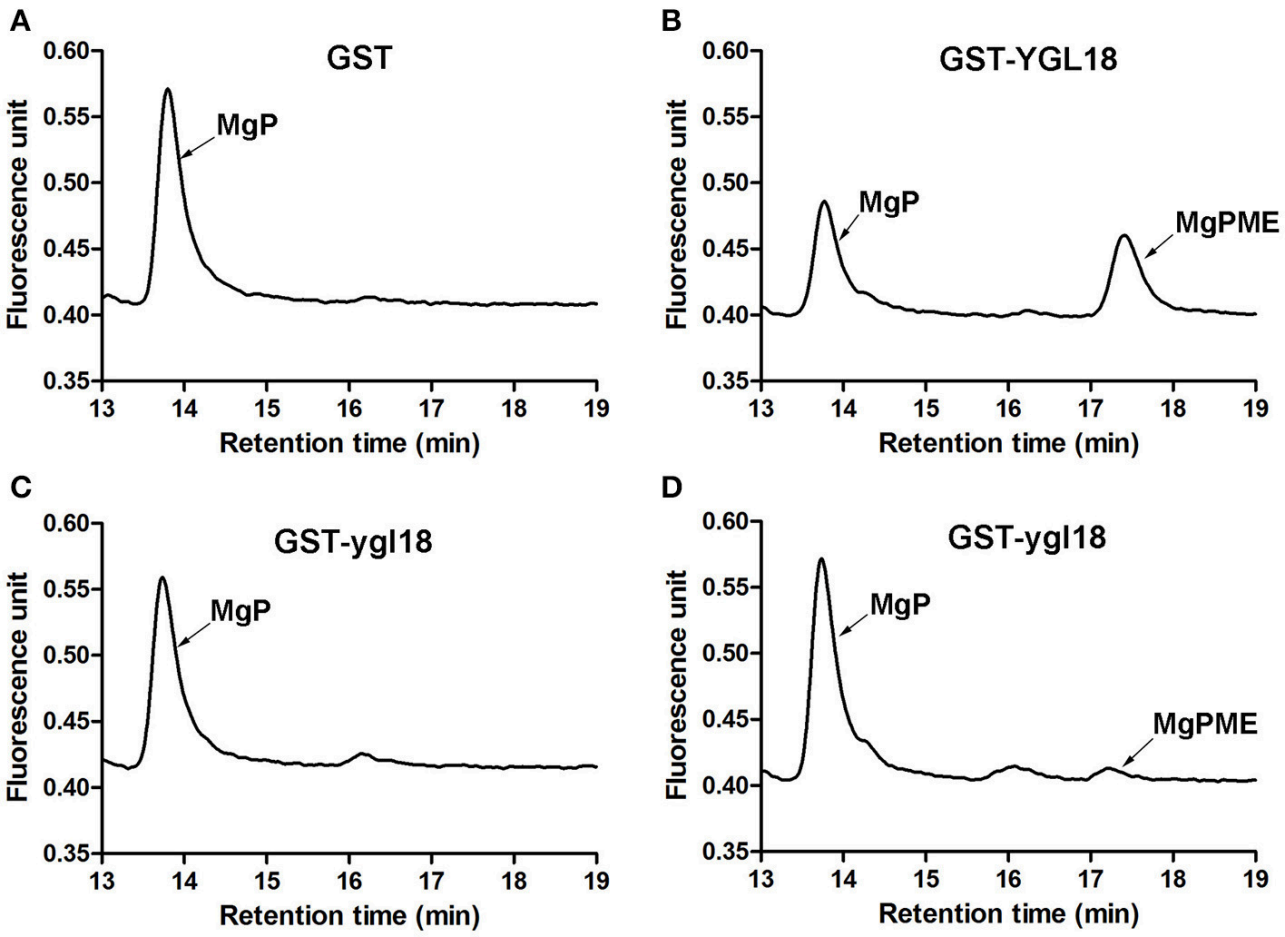
ChlM enzymatic activities of YGL18 and ygl18 based on HPLC. The enzymatic assays were conducted by adding different purified proteins: **(A)** GST (0.6 μM); **(B)** GST-YGL18 (0.6 μM); **(C)** GST-ygl18 (0.6 μM); **(D)** GST-ygl18 (2.4 μM). The arrows indicate the peak positions of the substrate MgP (about 13.7 min) and the product MgPME (about 17.3 min), which are distinguished by comparing with the standard curve (Figure S2). Different letters indicate values are statistically different based on a one-way ANOVA analysis.

In the pathway of chlorophyll synthesis, ChlM catalyzes the formation of MgPME from MgP (Beale, 2005; Richter et al., 2013). In this study, contents of MgPME and MgP in the enzymatic assay were determined by HPLC. After the enzymatic progression, the synthesis of MgPME from MgP was observed with the GST-YGL18 protein (Figure 6B), but not with the GST control (Figure 6A). Interestingly, GST-ygl18 failed to catalyze the enzymatic reaction at first (Figure 6C). However, when a 4 fold GST-ygl18 amount was added into the reaction, a small quantity of MgPME was produced (Figure 6D). These results suggested that the YGL18 protein indeed performs ChlM enzymatic activity, but the mutation of YGL18 to ygl18 substantially weakens this enzymatic function.

**Figure 7:**
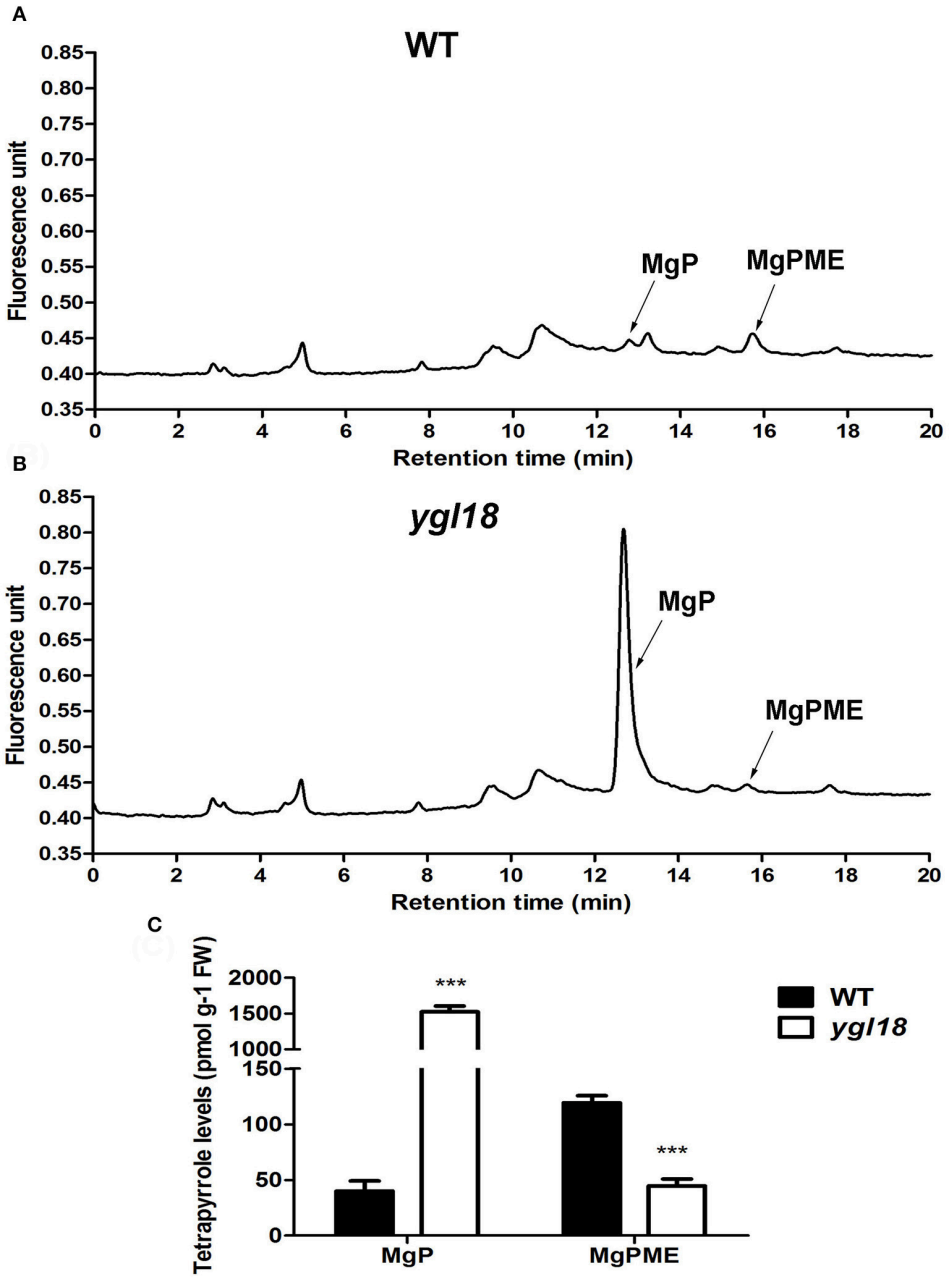
HPLC analysis of MgP and MgPME contents from the seedling leaves of wild-type and *ygl18* plants. **(A,B)** Representative chromatograms of the extracts from wild-type **(A)** and *ygl18* **(B)** plants (28°C, 8 h light condition, 150 μmol m^−2^ s^−1^). The arrows indicate the peak positions of MgP (about 12.7 min) and MgPME (about 15.8 min), which are distinguished by comparing with the standard curve (Figure S3). **(C)** Quantification of MgP and MgPME contents. FW, fresh weight. Data represent the mean ± SD of three biological replicates (Student's *t*-test: ^***^P < 0.0005). Different letters indicate values are statistically different based on a one-way ANOVA analysis.

To identify the attenuated ChlM function in the *ygl18* mutant, the MgPME and MgP contents were further measured in 15-day-old wild-type and *ygl18* plant leaves (28°, 8 h light) by HPLC (Figures 7A,B). As expected, the MgP content of 1,524.03 ± 81.28 pmol g^−1^ FW in *ygl18* greatly exceeded that of 39.96 ± 9.16 pmol g^−1^ FW in the wild type; by contrast, the MgPME content of 44.65 ± 6.13 pmol g^−1^ FW in *ygl18* was almost 3 fold less than that of 119.33 ± 6.44 pmol g^−1^ FW in wild type (Figure 7C). These results supported the fact that the mutation of YGL18 to ygl18 makes the ChlM enzymatic function notably weakened, thus leading to the accumulation of the substrate MgP and the reduction of the product MgPME in the *ygl18* mutant leaves.

### *YGL18* is required for light-dependent chlorophyll synthesis during the greening of etiolated plants

**Figure 8:**
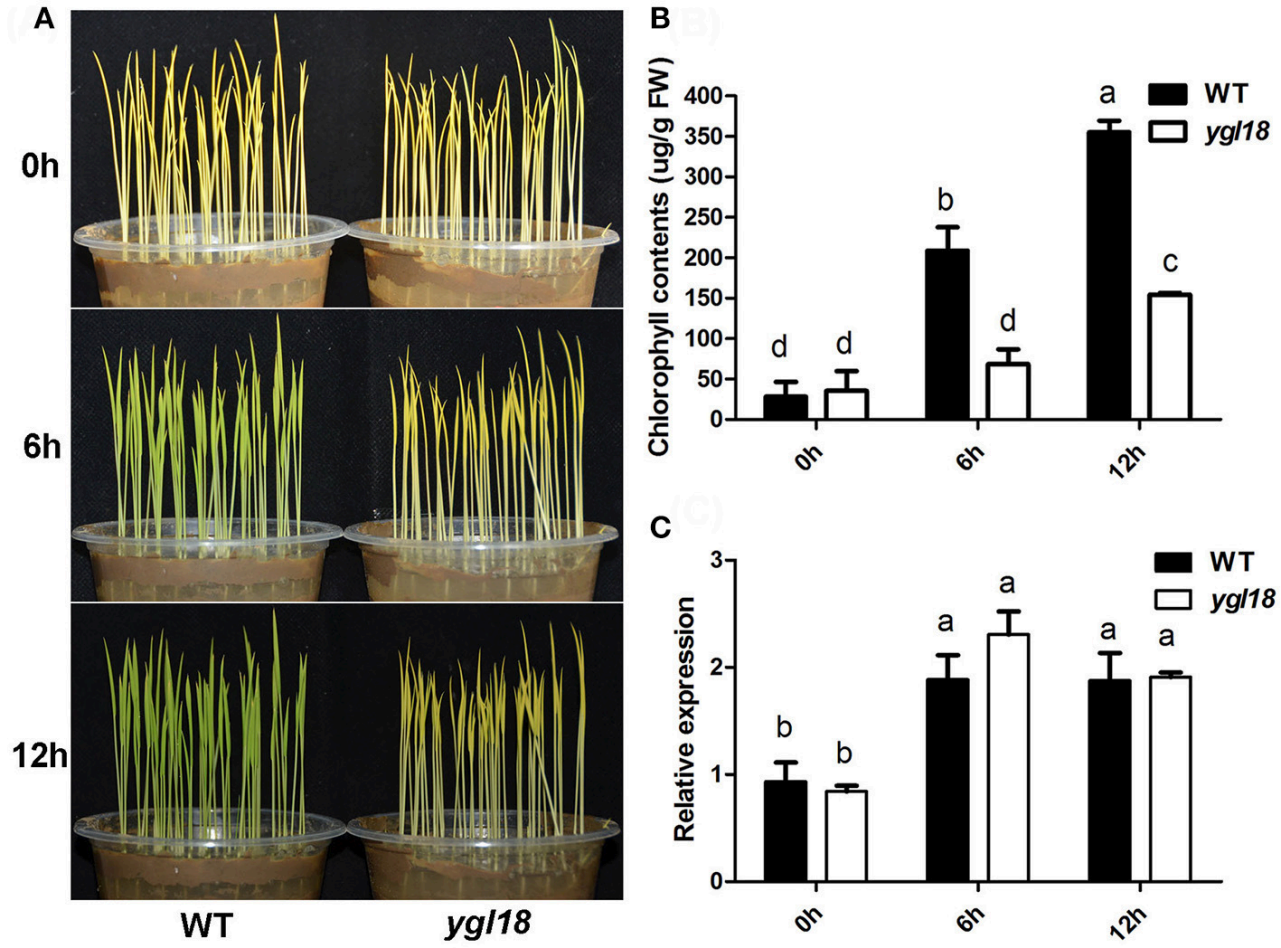
Expression levels of *YGL18* during the greening of etiolated seedlings. The 6-day-old etiolated seedlings of the wild type and *ygl18* were exposed to light (150 μmol m^−2^ s^−1^) for 0–12 h. **(A)** Comparison of the greening speed between the wild-type and *ygl18* seedlings. **(B)** Chlorophyll contents in the wild-type and *ygl18* seedlings during greening. **(C)** Changes in transcript levels of *YGL18* in the wild-type and *ygl18* seedlings during greening. In **(B,C)**, data represent the mean ± SD of three biological replicates (one-way ANOVA analysis); FW, fresh weight. Different letters indicate values are statistically different based on a one-way ANOVA analysis.

During the greening of etiolated plants, not every gene in the chlorophyll-synthesis pathway is required for light-dependent chlorophyll synthesis to occur. Previous studies have showed that *PGL* (*OsCAO1*) is required but *FGL* (*OsPORB*) is not essential for the process (Sakuraba et al., 2013; Yang et al., 2016). This differentiation prompted us to investigate the contribution of *YGL18* to chlorophyll synthesis. When 6-day-old etiolated wild-type and *ygl18* seedlings were exposed to light, the former quickly turned green, whereas the latter remained etiolated (Figure 8A). Correspondingly, chlorophyll contents increased rapidly in the etiolated wild-type seedlings, but this happened very slowly in the etiolated *ygl18* seedlings (Figure 8B). Further examination of the *YGL18* gene expression revealed it was rapidly upregulated in etiolated wild-type and *ygl18* seedlings after illumination (Figure 8C). Together, these results suggested that *YGL18* plays an essential role in the light-dependent chlorophyll synthesis during the greening of etiolated plants.

### *YGL18* is required for photoperiod-regulated chlorophyll synthesis

**Figure 9:**
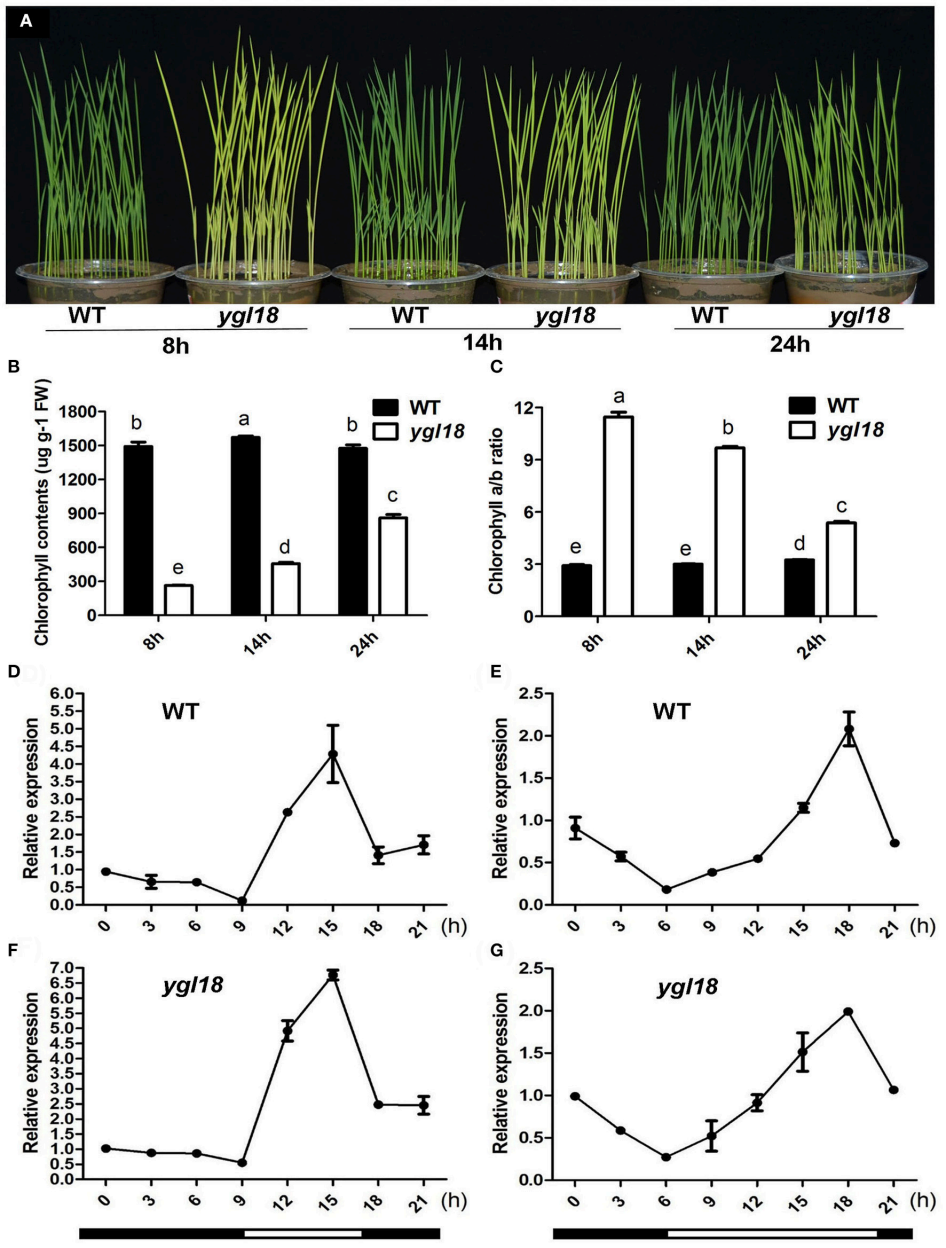
Photoperiod-dependent chlorophyll synthesis in *ygl18* leaves. **(A)** Leaf phenotypes of the 12-day-old wild-type and *ygl18* seedlings under short-day (8 h), long-day (14 h) and continuous (24 h) light conditions. **(B)** Chlorophyll contents of plant leaves in **(A)**. FW, fresh weight. **(C)** Chlorophyll *a*/*b* ratio of the plant leaves in **(A)**. In **(B,C)**, data represent the mean ± SD of four biological replicates (one-way ANOVA analysis). **(D–G)** Daily expression patterns of *YGL18* in the wild-type and *ygl18* seedling leaves under the short-day (**D**,**F**, 8 h light: 9:00 to 17:00.) and the long-day conditions (**E**,**G**, 14 h light: 6:00 to 20:00.). Leaf samples were harvested at 3 h intervals. Data represent the mean ± SD of two biological replicates. Different letters indicate values are statistically different based on a one-way ANOVA analysis.

Surprisingly, the *ygl18* plants showed different leaf colors under varied photoperiod conditions. When the seedlings grew under the short-day (8 h), long-day (14 h) and continuous (24 h) light conditions, the *ygl18* leaf colors were yellow, yellow-green, and pale green, respectively (Figure 9A). The corresponding chlorophyll contents were measured subsequently. They were lowest for the short-day but increased gradually for the long-day and continuous light conditions in *ygl18* leaves (Figure 9B). Meanwhile, the chlorophyll *a*/*b* ratio was highest for the short-day but decreased gradually for the long-day and continuous light conditions in *ygl18* leaves (Figure 9C). In addition, we tested the daily expression patterns of the *YGL18* gene in wild-type and *ygl18* seedling leaves under the short-day and long-day conditions. Under both conditions, the *YGL18* expression levels in the wild-type and *ygl18* seedling leaves were upregulated by light yet downregulated by dark (ons, the *ygl18* leaf colors were yellow, yellow-green, and pale green, respectively (Figures 9D–G). Taken together, these results suggested that the *YGL18* gene is required for photoperiod-regulated chlorophyll synthesis.

### *YGL18* is required for plant growth and survival under high light conditions

**Figure 10:**
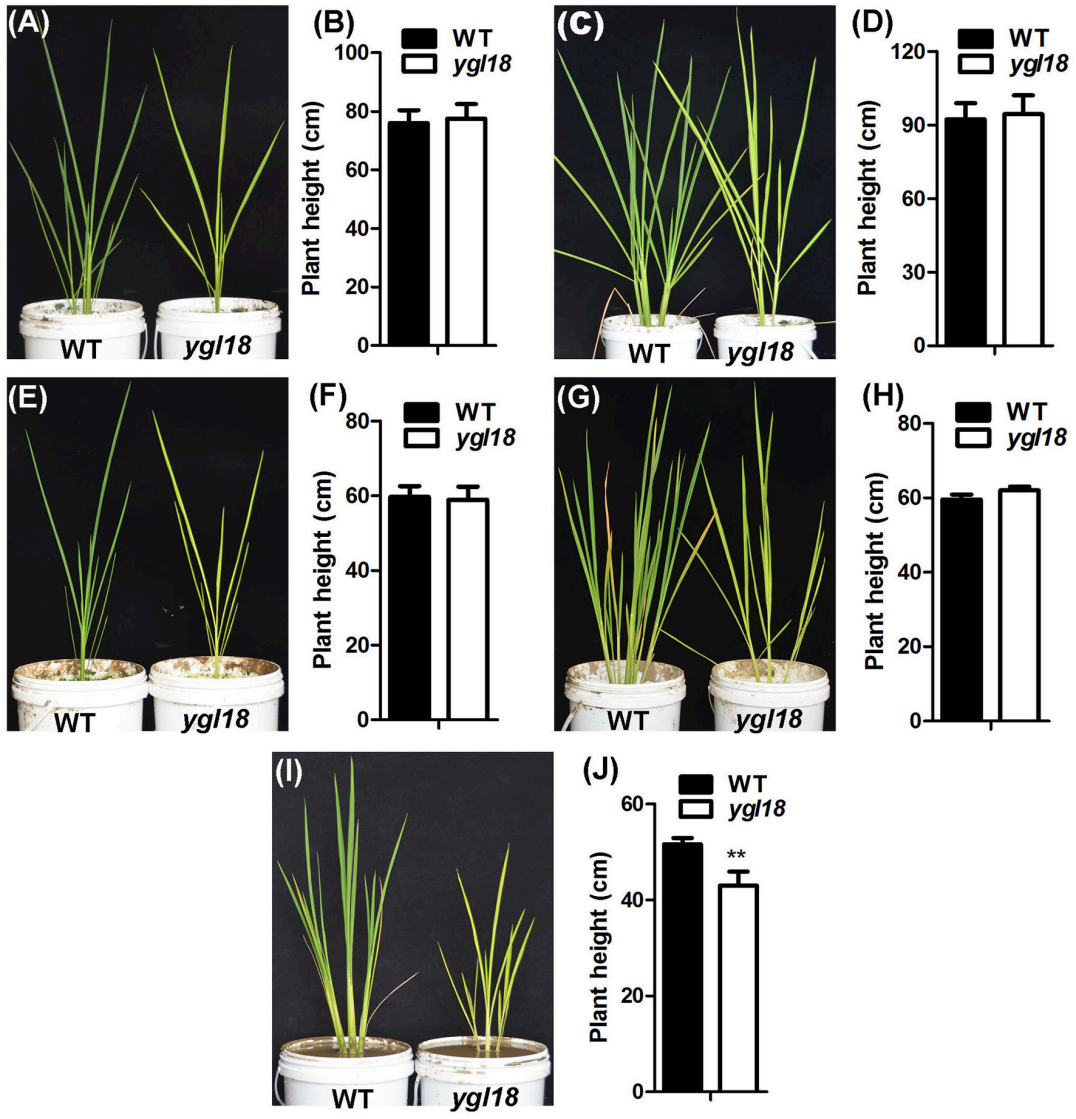
Growth of the wild-type and *ygl18* plants under different experimental conditions in the plant growth chamber. Fifteen-day-old seedlings (28/22°C, 14/10 h, light/dark, 190 μmol m^−2^ s^−1^) were used for the following experimental conditions. **(A)** Plants grown for about 60 days under the condition of 26/19°C, 14/10 h, 190 μmol m^−2^ s^−1^. **(B)** Plant height under the condition in **(A)**. **(C)** Plants grown for about 60 days under the condition of 31/22°C, 14/10 h, 190 μmol m^−2^ s^−1^. **(D)** Plant height under the condition in **(C)**. **(E)** Plants grown for about 30 days under the condition of 35/22°C, 14/10 h, 190 μmol m^−2^ s^−1^. **(F)** Plant height under the condition in **(E)**. **(G)** Plants grown for about 30 days under the condition of 31/22°C, 14/10 h, 500 μmol m^−2^ s^−1^. **(H)** Plant height under the condition in **(G)**. **(I)** Plants grown for about 23 days under the condition of 31/22°C, 14/10 h, 900 μmol m^−2^ s^−1^. **(J)** Plant height under the condition in **(I)**. Data represent the mean ± SD of three independent plants (Student's *t*-test: ^**^P < 0.005).

The *ygl18* mutants showed severely stunted growth, and even death, when planted in Wuhan of Hubei province, but they grew similarly to the wild type when planted in Lingshui of Hainan province (Figures 1D,E). It would be interesting to know the reason for this growth difference. Under natural field conditions, a longer day length, higher temperature and higher solar radiation occurred at the planting time in Wuhan than in Lingshui (Figure S6). Accordingly, we explored the growth status of the wild-type and *ygl18* plants under different experimental conditions in the plant growth chamber. When the wild-type and *ygl18* plants were cultivated under a 14-h light condition (190 μmol m^−2^ s^−1^, 26/19°C for light/dark), mimicking the day length in Wuhan, the *ygl18* mutant grew to a similar height as the wild type (Figures 10A,B). Based on this growth condition, the growth temperature was further raised to 31/22°C and 35/22°C (14-h light, 190 μmol m^−2^ s^−1^), but the *ygl18* plants maintained equivalent growth with the wild type (Figures 10C–F). Next, we tried to enhance the light intensity. The *ygl18* plants still grew to a similar height as the wild type when the light intensity was set to 500 μmol m^−2^ s^−1^ (14-h light, 31/22°C) (Figures 10G,H). However, when the light intensity was enhanced to 900 μmol m^−2^ s^−1^ (14-h light, 31/22°C), the *ygl18* plants did not grow as well as the wild type (Figures 10I,J). Therefore, it is inferred that it was the higher light intensity that caused the retarded growth of *ygl18* in Wuhan, but neither its longer day length nor higher temperature.

**Figure 11:**
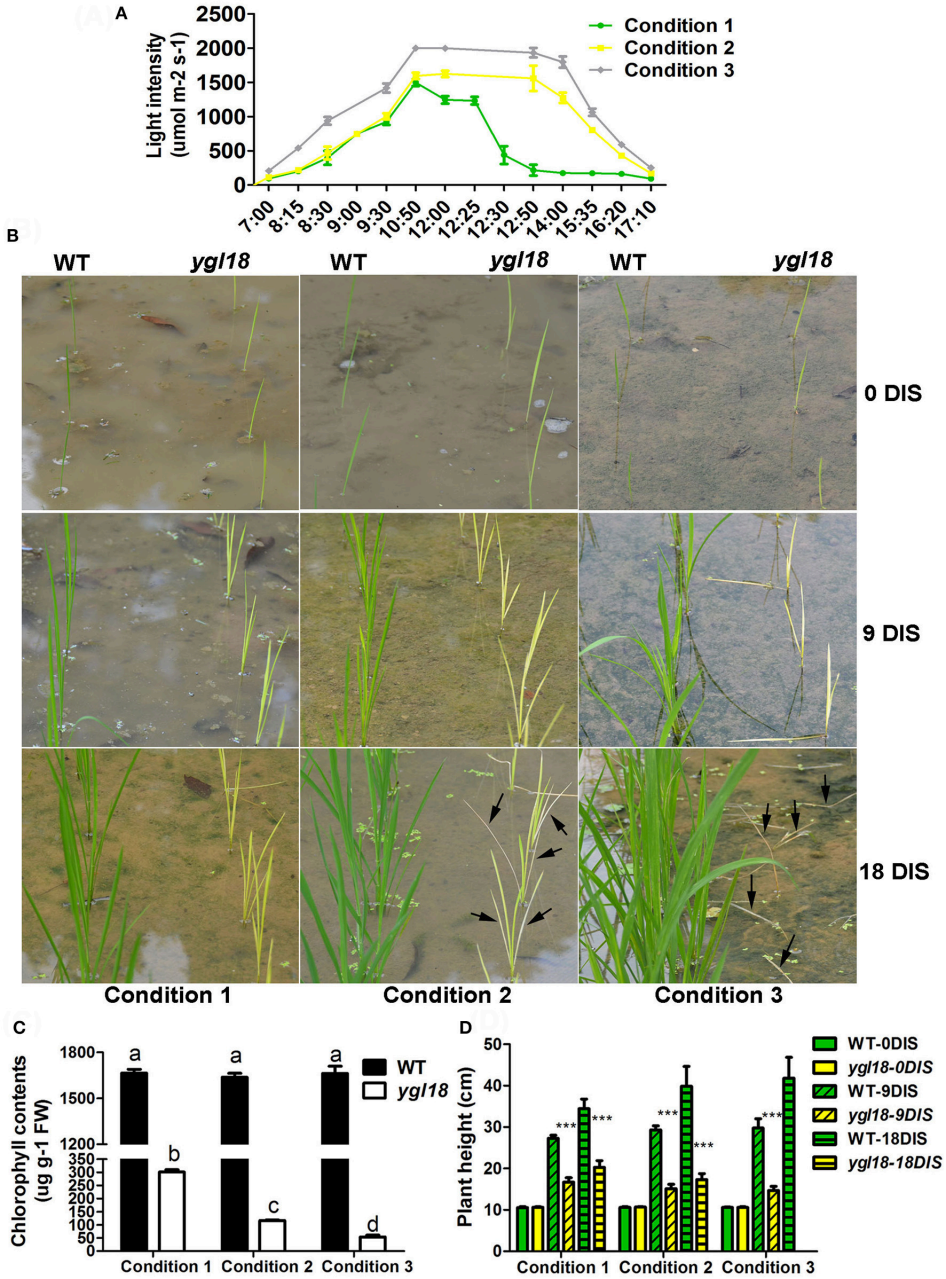
Growth of the wild-type and *ygl18* plants under different light conditions in the natural field. **(A)** Three different light conditions were generated by the solar motion and the tree shadow. Condition 1 had about 4 h' time in sunlight (8:30–12:30), about 3 h' light intensity over 1,000 μmol m^−2^ s^−1^, and a peak light intensity of about 1,500 μmol m^−2^ s^−1^. Condition 2 had about 8 h' time in sunlight (8:30–16:20), about 5 h' light intensity over 1,000 μmol m^−2^ s^−1^, and a peak light intensity of about 1,600 μmol m^−2^ s^−1^. Condition 3 had about 8 h' time in sunlight (8:15–16:20), about 7 h' light intensity over 1,000 μmol m^−2^ s^−1^, and a peak light intensity of over 2,000 μmol m^−2^ s^−1^. **(B)** Growth status of the wild-type and *ygl18* plants under above three light conditions. Eight-day-old seedlings were planted in the fields and encountered one cloudy day and subsequent 18 sunny days (condition 1, 2, and 3). Plants were observed after 0, 9, and 18 days in sunlight (DIS). The black arrows indicate the withered leaves. **(C)** Chlorophyll contents of the wild-type and *ygl18* plant leaves at 9 DIS. Data represent the mean ± SD of three biological replicates (one-way ANOVA analysis); FW, fresh weight. **(D)** Plant height of the wild-type and *ygl18* plants at 0, 9, and 18 DIS under above three conditions. Data represent the mean ± SD of over eight independent plants (Student's *t*-test: ^***^P < 0.0005). ND, not detected for dead plants. Different letters indicate values are statistically different based on a one-way ANOVA analysis.

We also explored the growth status of wild-type and *ygl18* plants under three different natural field conditions. These three conditions in the planting field were emerged by the solar motion combining with tree shadow, resulting in the sunlight time and light intensity lowest for condition 1, moderate for condition 2, and highest for condition 3 (Figure 11A). Eight-day-old seedlings were simultaneously planted in the fields under these three light conditions (Figure 11B). Then, they encountered one cloudy day and subsequent 18 sunny days. After 9 days in sunlight (DIS), the *ygl18* mutant showed yellow-green leaves under condition 1, yellow leaves under condition 2, and yellow-tending-to-white leaves under condition 3 (Figure 11B). Correspondingly, under conditions 1, 2, and 3, the chlorophyll contents were gradually decreased in *ygl18* but remained steady and stable in wild-type seedlings after nine DIS (Figure 11C). Surprisingly, after 18 DIS, the *ygl18* mutants showed yellow leaves under condition 1, but yellow-tending-to-white and partially withered leaves under condition 2, and totally withered and dead leaves under condition 3 (Figure 11B). In addition, because of the high light intensity, the growth of all the *ygl18* plants was markedly reduced under these three conditions, as we expected (Figure 11D).

Taken together, *ygl18* showed impeded growth under high light intensity. When the light intensity was sufficiently high, the *ygl18* seedlings were more susceptible to die at this light intensity or after longer exposure to it. It is suggested that *YGL18* plays an essential role in plant growth and survival under high light conditions.

## Discussion

### The *YGL18* gene is cloned as the *ChlM* gene in the monocotyledonous plant—rice

The *ChlM* gene is one of the key genes in the chlorophyll synthesis pathway. Its mutants have ever only been found in *Arabidopsis* (Pontier et al., 2007) and *Chlamydomonas reinhardtii* (Meinecke et al., 2010). And its enzymatic activities have been well studied in *Synechocystis* sp. PCC6803 (Shepherd et al., 2003; Shepherd and Hunter, 2004), tobacco (Alawady et al., 2005), and *Arabidopsis* (Block et al., 2002; Richter et al., 2013, 2016). To date, however, no *ChlM* gene has yet been identified and studied in monocotyledonous plants. In the present study, a spontaneous yellow-green-leaf rice mutant, *ygl18*, was successfully isolated (Figure 1), and the *YGL18* gene was cloned (Figure 2), that encodes a protein homologous with the AtChlM (Figure 5 and Figure S4). The recombinant YGL18 protein expressed in E. *coli* was shown to perform ChlM enzymatic activity (Figure 6). Therefore, we succeeded in identifying a *ChlM* gene in a monocotyledonous plant.

### The rice YGL18 protein is a functionally conserved ChlM protein, and the substitution of a conserved leu to Phe in ygl18 starkly weakens its function

The amino acid residues of YGL18 and other ChlM orthologues from different photosynthetic organisms are highly conserved (Figure 5). These conserved sequences belong to the conserved protein domain “BchM-ChlM” (TIGR02021), which represents the *S*-adenosylmethionine-dependent O-methyltransferase responsible for the methylation of MgP (Marchler-Bauer et al., 2015). These conserved amino acid residues may be essential for the ChlM function. A missense mutation of *chlM-1* in *Chlamydomonas reinhardtii* made these mutants incapable of accumulating chlorophyll (Meinecke et al., 2010), where the mutation resulted in a replacement of a leucine (Leu) by a proline (Pro) residue at position 270 in CrChlM, meanwhile, Leu at this particular position remained completely conserved in our analysis (Figure 5). In this study, the Leu at position 225 in the YGL18 protein is also originally a fully conserved amino acid residue among the analyzed organisms, but it is substituted by Phe in the ygl18 protein (Figure 5). It is speculated that this Leu may play an important role for the OsChlM protein function. Previous study has indicated that the amino acid of ChlM proteins corresponding to the Leu at position 225 in the YGL18 protein is involved in binding to the cofactor SAM for ChlM proteins in different organisms (Chen et al., 2014). In addition, it was recently proposed that the NADPH-dependent thioredoxin reductase C has a regulatory impact on the redox status of conserved cysteine residues of ChlM, thus altering the ChlM activity (Richter et al., 2013). Further research has identified three conserved cysteine residues essential for the catalytic function and the redox-dependent activation of the Arabidopsis ChlM (Richter et al., 2016). These three cysteine residues are also conserved in the YGL18 protein (Figure 5), implying their possible functions on the redox regulation of ChlM enzymatic activity in rice. Interestingly, the third cysteine is just located at the left neighboring site of Leu 225 of YGL18 (Figure 5), meaning that the Leu at position 225 is also probably involved in redox regulation of ChlM enzymatic activity. Therefore, it is plausible that the amino-acid substitution of Leu to Phe in the ygl18 protein may impact the ChlM activity by changing its binding or catalyzing ability to SAM.

ChlM catalyzes the formation of MgPME from MgP in the chlorophyll synthesis pathway (Richter et al., 2013). In this study, the YGL18 protein was also verified to perform the ChlM activity (Figure 6B), just as the ChlM proteins do in *Synechocystis* sp. PCC6803 (Shepherd et al., 2003; Shepherd and Hunter, 2004), *Arabidopsis* (Block et al., 2002), and tobacco (Alawady et al., 2005). This clearly confirms that the *YGL18* gene is the corresponding *ChlM* gene in rice. The *in vitro* assay revealed that the ChlM enzymatic activity of the ygl18 protein was greatly reduced, with the slight synthesis of MgPME from MgP requiring a large amount of the ygl18 protein (Figures 6C,D). Further, given that the MgPME content could be measured in the *ygl18* leaves (Figures 7B,C), this supports the conclusion that the single amino acid exchange from Leu to Phe in ygl18 did not completely negate the protein's biological function. Most possibly, it is just the remained slight activity of the ygl18 protein that results in the characteristics of *ygl18* mutant on chlorophyll synthesis and plant growth, as discussed later.

### The rice *YGL18 (ChlM)* gene is required for light-dependent and photoperiod-regulated chlorophyll synthesis

The *YGL18* gene is mainly expressed in green tissues, especially in leaves (Figure 3), where the YGL18 protein is localized in the chloroplasts (Figure 4). These results are all consistent with the fact that the *ChlM* gene encodes one of the key enzymes in the chlorophyll synthesis pathway. Among the cloned genes in the chlorophyll synthesis pathway, the *PGL* (*OsCAO1*) gene was identified as required for light-dependent chlorophyll synthesis during the greening of the etiolated plants (Yang et al., 2016), but the *FGL* (*OsPORB*) gene was verified as not required for this biological process (Sakuraba et al., 2013). In this study, the *YGL18* (*ChlM*) gene was clearly essential for light-dependent chlorophyll synthesis during the greening of the etiolated WT and *ygl18* plants (Figure 8). Impact of photoperiod on chlorophyll synthesis is rarely reported. More chlorophyll contents were accumulated in the *ygl18* leaves when grown under a longer light application time (Figures 9A,B). Similar phenomena were found from the *chlB* knockout mutant in liverwort (*Marchantia polymorpha* L.), where it was suggested that *chlB* was required for chlorophyll biosynthesis under short photoperiod (Ueda et al., 2014). Nevertheless, there may be a different explanation for this phenomenon in *ygl18*. The ygl18 protein did not completely cancel the protein function, and a large amount of the ygl18 protein could catalyze the formation of a small quantity of MgPME (Figure 6D). In addition, the circadian expression patterns of the *YGL18* gene revealed that it was up-regulated by light yet down-regulated by dark conditions (Figures 9D–G). As a result, under longer light conditions, the *ygl18* mutant possibly produced more ygl18 proteins for the enzymatic catalyzation, and thus eventually accumulated more chlorophyll in its leaves. Taken together, the results strongly suggested that the *YGL18* (*ChlM*) gene plays an essential role in light-dependent and photoperiod-regulated chlorophyll synthesis.

### The rice *YGL18 (ChlM)* gene is essential for plant growth and survival under high light conditions

Scarce are studies that pay in-depth attention to the effect on plant growth from functional genes in the chlorophyll synthesis pathway. It was surprising that *ygl18* showed severely retarded growth, and even death, in the paddy field in Wuhan, but near-normal growth in Lingshui (Figure 1). When the wild-type and *ygl18* plants were cultivated in the plant growth chamber under different temperature and light conditions, it was the high light intensity, but not the long day length or the high temperature, which impaired the *ygl18* growth (Figure 10). Field experiments with different sunlight time and light intensity further showed that the *ygl18* plants were more prone to death under a higher light intensity and a longer exposure time to it (Figure 11). Effects of light intensity upon *chlM* mutants have also been reported for other species. In *Chlamydomonas reinhardtii, chlM-1* has an amino-acid substitution, while *chlM-2* do not produce the ChlM protein; consequently, *chlM-1* can survive under the light intensity of 45 μmol m^−2^ s^−1^, but not under a higher light intensity of 500 μmol m^−2^ s^−1^, while *chlM-2* can only weakly survive under 1.6 μmol m^−2^ s^−1^ of light (Meinecke et al., 2010). The *Arabidopsis chlM* mutant lacks the *ChlM* mRNA and ChlM protein because of the T-DNA insertion; hence, its mutant seedlings turned white and stopped growing after cotyledon development when germinated under the light intensity of 70 μmol m^−2^ s^−1^ (Pontier et al., 2007). Apparently, the complete loss of ChlM function is lethal for plants to grow under well-lit conditions, while an impaired ChlM function makes the plants survival only under low light intensity conditions. Meanwhile, MgP, the substrate of ChlM, notably accumulated in *Chlamydomonas reinhardtii* and *Arabidopsis chlM* mutants, as described. Similarly, we found that MgP also remarkably accumulated in the rice *ygl18* mutants (Figures 7B–C). However, rice mutants, which were related to genes in the chlorophyll synthesis pathway, such as *ygl7* (Deng et al., 2014), *ygl1* (Wu et al., 2007), and *pgl* (Yang et al., 2016), grew only slightly lower than their wild type, and these mutants still contained plenty of chlorophyll thus showed near-green leaves. It was also the fact that the intermediate metabolites of chlorophyll synthesis reportedly did not substantially accumulate (about 2-fold for MgP, and <2-fold for chlide, the substrate of YGL1) in *ygl1* mutants. Considering these studies, it would appear that fluent synthesis of chlorophyll is essential for functional plant growth. The accumulation of the intermediate metabolites, like MgP, is probably the key factor to influence plant growth under high light conditions. When accumulated, such intermediate metabolites may induce oxidative damages under light irradiation, while photoautotrophic growth relying on chlorophyll synthesis may support plant resistance to this damages. It is speculated that a dynamic equilibrium between the damages induced by accumulated MgP and resistance originating from the leaky synthesis of chloroph1yll is critical for the growth of *ygl18* plants. To completely elucidate this scientific issue, a further detailed study will be needed.

## Conclusion

In conclusion, this study identified the *ChlM* gene in rice plants. It is expressed mainly in the leaf tissue and its protein product, is localized in the chloroplasts to perform the biological roles. The enzymatic activity of ChlM was verified. *ChlM* is required for light-dependent and photoperiod-regulated chlorophyll synthesis. And also, *ChlM* plays an essential role in light intensity-related plant growth and survival dynamics.

## Author contributions

ZW designed and participated in all the experimental procedures, performed the data analysis and also drafted the manuscript; XH participated in the gene cloning and complementary experiments; KH performed the gene expression experiments; YW helped to prepare the figures and did partial writing; XW and SD participated in planting the rice materials; YL participated in the vector construction; DH and KC participated in the sample preparation; BA participated in the figure preparation; YsL supervised the study and critically revised the manuscript. All authors read and approved the final manuscript.

### Conflict of interest statement

The authors declare that the research was conducted in the absence of any commercial or financial relationships that could be construed as a potential conflict of interest.

## Abbreviations

CDS: coding sequence
ChlM: magnesium protoporphyrin IX methyltransferase
DIS: days in sunlight
FW: fresh weigh
GST: glutathione-*S*-transferase
MgP: magnesium protoporphyrin IX
MgPME: magnesium protoporphyrin IX monomethylester
qRT-PCR: quantitative real-time reverse transcription polymerase chain reaction
SAM: *S*-adenosylmethionine
*ygl18*: *yellow-green leaf 18*
YFP: yellow fluorescent protein.

## References

[B1] Alawady A. E. Grimm B. (2005). Tobacco Mg protoporphyrin IX methyltransferase is involved in inverse activation of Mg porphyrin and protoheme synthesis. Plant J. 41, 282–290. 10.1111/j.1365-313X.2004.02291.x 15634204

[B2] Alawady A. Reski R. Yaronskaya E. Grimm B. (2005). Cloning and expression of the tobacco CHLM sequence encoding Mg protoporphyrin IX methyltransferase and its interaction with Mg chelatase. Plant Mol. Biol. 57, 679–691. 10.1007/s11103-005-1427-8 15988563

[B3] Beale S. I. (2005). Green genes gleaned. Trends Plant Sci. 10, 309–312. 10.1016/j.tplants.2005.05.005 15951223

[B4] Block M. A. Tewari A. K. Albrieux C. Marechal E. Joyard J. (2002). The plant S-adenosyl-L-methionine:Mg-protoporphyrin IX methyltransferase is located in both envelope and thylakoid chloroplast membranes. Eur. J. Biochem. 269, 240–248. 10.1046/j.0014-2956.2001.02643.x 11784318

[B5] Chen X. Wang X. Feng J. Chen Y. Fang Y. Zhao S.. (2014). Structural insights into the catalytic mechanism of Synechocystis magnesium protoporphyrin IX O-methyltransferase (ChlM). J. Biol. Chem. 289, 25690–25698. 10.1074/jbc.M114.584920 25077963 PMC4162172

[B6] Czarnecki O. Grimm B. (2012). Post-translational control of tetrapyrrole biosynthesis in plants, algae, and cyanobacteria. J. Exp. Bot. 63, 1675–1687. 10.1093/jxb/err437 22231500

[B7] Deng X. J. Zhang H. Q. Wang Y. He F. Liu J. L. Xiao X.. (2014). Mapped clone and functional analysis of leaf-color gene Ygl7 in a rice hybrid (*Oryza sativa* L. ssp. indica). PLoS ONE 9:e99564. 10.1371/journal.pone.0099564 24932524 PMC4059691

[B8] Fromme P. Melkozernov A. Jordan P. Krauss N. (2003). Structure and function of photosystem I: interaction with its soluble electron carriers and external antenna systems. FEBS Lett. 555, 40–44. 10.1016/S0014-5793(03)01124-4 14630316

[B9] Grossman A. R. Bhaya D. Apt K. E. Kehoe D. M. (1995). Light-harvesting complexes in oxygenic photosynthesis: diversity, control, and evolution. Annu. Rev. Genet. 29, 231–288. 10.1146/annurev.ge.29.120195.001311 8825475

[B10] Jung K. H. Hur J. Ryu C. H. Choi Y. Chung Y. Y. Miyao A.. (2003). Characterization of a rice chlorophyll-deficient mutant using the T-DNA gene-trap system. Plant Cell Physiol. 44, 463–472. 10.1093/pcp/pcg064 12773632

[B11] Lee S. Kim J. H. Yoo E. S. Lee C. H. Hirochika H. An G. (2005). Differential regulation of chlorophyll a oxygenase genes in rice. Plant Mol. Biol. 57, 805–818. 10.1007/s11103-005-2066-9 15952067

[B12] Lichtenthaler H. K. (1987). Chlorophylls and carotenoids: pigments of photosynthetic biomembranes. Meth. Enzymol. 148, 350–382. 10.1016/0076-6879(87)48036-1

[B13] Marchler-Bauer A. Derbyshire M. K. Gonzales N. R. Lu S. Chitsaz F. Geer L. Y.. (2015). CDD: NCBI's conserved domain database. Nucleic Acids Res. 43, D222–D226. 10.1093/nar/gku1221 25414356 PMC4383992

[B14] Meinecke L. Alawady A. Schroda M. Willows R. Kobayashi M. C. Niyogi K. K.. (2010). Chlorophyll-deficient mutants of Chlamydomonas reinhardtii that accumulate magnesium protoporphyrin IX. Plant Mol. Biol. 72, 643–658. 10.1007/s11103-010-9604-9 20127142 PMC2837180

[B15] Mochizuki N. Tanaka R. Tanaka A. Masuda T. Nagatani A. (2008). The steady-state level of Mg-protoporphyrin IX is not a determinant of plastid-to-nucleus signaling in *Arabidopsis*. Proc. Natl. Acad. Sci. U.S.A. 105, 15184–15189. 10.1073/pnas.0803245105 18818313 PMC2567512

[B16] Nagata N. Tanaka R. Satoh S. Tanaka A. (2005). Identification of a vinyl reductase gene for chlorophyll synthesis in *Arabidopsis thaliana* and implications for the evolution of *Prochlorococcus* species. Plant Cell 17, 233–240. 10.1105/tpc.104.027276 15632054 PMC544501

[B17] Pontier D. Albrieux C. Joyard J. Lagrange T. Block M. A. (2007). Knock-out of the magnesium protoporphyrin IX methyltransferase gene in *Arabidopsis*. J. Biol. Chem. 282, 2297–2304. 10.1074/jbc.M610286200 17135235 PMC2408936

[B18] Richter A. S. Peter E. Rothbart M. Schlicke H. Toivola J. Rintamaki E. (2013). Post-translational influence of NADPH-dependent thioredoxin reductase C on enzymes in tetrapyrrole synthesis. Plant Physiol. 162, 63–73. 10.1104/pp.113.217141 23569108 PMC3641230

[B19] Richter A. S. Wang P. Grimm B. (2016). Arabidopsis Mg-Protoporphyrin IX Methyltransferase activity and redox regulation depend on conserved cysteines. Plant Cell Physiol. 57, 519–527. 10.1093/pcp/pcw007 26759408

[B20] Sakuraba Y. Rahman M. L. Cho S. H. Kim Y. S. Koh H. J. Yoo S. C.. (2013). The rice faded green leaf locus encodes protochlorophyllide oxidoreductase B and is essential for chlorophyll synthesis under high light conditions. Plant J. 74, 122–133. 10.1111/tpj.12110 23289852

[B21] Sheng Z. Lv Y. Li W. Luo R. Wei X. Xie L.. (2017). Yellow-Leaf 1 encodes a magnesium-protoporphyrin IX monomethyl ester cyclase, involved in chlorophyll biosynthesis in rice (*Oryza sativa* L.). PLoS ONE 12:e0177989. 10.1371/journal.pone.0177989 28558018 PMC5448749

[B22] Shepherd M. Hunter C. N. (2004). Transient kinetics of the reaction catalysed by magnesium protoporphyrin IX methyltransferase. Biochem. J. 382(Pt 3), 1009–1013. 10.1042/BJ20040661 15239672 PMC1133978

[B23] Shepherd M. Reid J. D. Hunter C. N. (2003). Purification and kinetic characterization of the magnesium protoporphyrin IX methyltransferase from *Synechocystis* PCC6803. Biochem. J. 371(Pt 2), 351–360. 10.1042/bj20021394 12489983 PMC1223276

[B24] Strand A. Asami T. Alonso J. Ecker J. R. Chory J. (2003). Chloroplast to nucleus communication triggered by accumulation of Mg-protoporphyrinIX. Nature 421, 79–83. 10.1038/nature01204 12511958

[B25] Sun C. Liu L. Tang J. Lin A. Zhang F. Fang J.. (2011). RLIN1, encoding a putative coproporphyrinogen III oxidase, is involved in lesion initiation in rice. J. Genet. Genomics 38, 29–37. 10.1016/j.jcg.2010.12.001 21338950

[B26] Ueda M. Tanaka A. Sugimoto K. Shikanai T. Nishimura Y. (2014). chlB requirement for chlorophyll biosynthesis under short photoperiod in *Marchantia polymorpha* L. Genome Biol. Evol. 6, 620–628. 10.1093/gbe/evu045 24586029 PMC3971596

[B27] Vandesompele J. De Preter K. Pattyn F. Poppe B. Van Roy N. De Paepe A.. (2002). Accurate normalization of real-time quantitative RT-PCR data by geometric averaging of multiple internal control genes. Genome Biol. 3:RESEARCH0034. 10.1186/gb-2002-3-7-research0034 12184808 PMC126239

[B28] Van Wilder V. De Brouwer V. Loizeau K. Gambonnet B. Albrieux C. Van Der Straeten D.. (2009). C1 metabolism and chlorophyll synthesis: the Mg-protoporphyrin IX methyltransferase activity is dependent on the folate status. New Phytol. 182, 137–145. 10.1111/j.1469-8137.2008.02707.x 19076298

[B29] Wang P. Gao J. Wan C. Zhang F. Xu Z. Huang X.. (2010). Divinyl chlorophyll(ide) a can be converted to monovinyl chlorophyll(ide) a by a divinyl reductase in rice. Plant Physiol. 153, 994–1003. 10.1104/pp.110.158477 20484022 PMC2899930

[B30] Wang X. Huang R. Quan R. (2017). Mutation in Mg-Protoporphyrin IX .monomethyl ester cyclase decreases photosynthesis capacity in rice. PLoS ONE 12:e0171118. 10.1371/journal.pone.0171118 28129387 PMC5271374

[B31] Wang Z. Wang Y. Hong X. Hu D. Liu C. Yang J.. (2015). Functional inactivation of UDP-N-acetylglucosamine pyrophosphorylase 1 (UAP1) induces early leaf senescence and defence responses in rice. J. Exp. Bot. 66, 973–987. 10.1093/jxb/eru456 25399020 PMC4321554

[B32] Wang Z. Wang Y. Yang J. Hu K. An B. Deng X.. (2016). Reliable selection and holistic stability evaluation of reference genes for rice under 22 different experimental conditions. Appl. Biochem. Biotechnol. 179, 753–775. 10.1007/s12010-016-2029-4 26940571

[B33] Wu Z. Zhang X. He B. Diao L. Sheng S. Wang J.. (2007). A chlorophyll-deficient rice mutant with impaired chlorophyllide esterification in chlorophyll biosynthesis. Plant Physiol. 145, 29–40. 10.1104/pp.107.100321 17535821 PMC1976586

[B34] Yang Y. Xu J. Huang L. Leng Y. Dai L. Rao Y.. (2016). PGL, encoding chlorophyllide a oxygenase 1, impacts leaf senescence and indirectly affects grain yield and quality in rice. J. Exp. Bot. 67, 1297–1310. 10.1093/jxb/erv529 26709310 PMC4762379

[B35] Yu C. Wang L. Chen C. He C. Hu J. Zhu Y.. (2014). Protoplast: a more efficient system to study nucleo-cytoplasmic interactions. Biochem. Biophys. Res. Commun. 450, 1575–1580. 10.1016/j.bbrc.2014.07.043 25026554

[B36] Zhang H. Li J. Yoo J. H. Yoo S. C. Cho S. H. Koh H. J.. (2006). Rice Chlorina-1 and Chlorina-9 encode ChlD and ChlI subunits of Mg-chelatase, a key enzyme for chlorophyll synthesis and chloroplast development. Plant Mol. Biol. 62, 325–337. 10.1007/s11103-006-9024-z 16915519

