# Supplementary material for: Impaired Magnesium Protoporphyrin IX Methyltransferase (ChlM) Impedes Chlorophyll Synthesis and Plant Growth in Rice
Source: Front Plant Sci. 2017 Sep 28;8:1694. doi: 10.3389/fpls.2017.01694 (PMC5626950; doi:10.3389/fpls.2017.01694)
Supplement: Supplementary file 1 [file Table1.PDF]

Table S1 List of PCR-based molecular markers used for map-based cloning of *YGL18*

Marker name	Forward primer	Reverse primer
Chr6mm0016	ACTCCCTCGTCTCTAGAGTTCTCC	GTTGGGAGTCTATCCCATCG
Chr6mm0129	CGTGCTATCACATGCAGAAGACC	GTTGCCGTCACCTGAATATAGGG
Chr6mm0287	ACACCACCATCAACGTACCAACC	AAGTCGAGAGGAAGAAGCCAAGG
Y11	TAAGACGAAAGCTCAAAAGTTG	CATGTCAAATATCACTAGGTAAGGTA
Y13	TACGCTTATTCGGATTACGAG	CTACCTCATTTTTTCCATTTCTTAC
Y15	GGGGTGTTTAGAGCAAGTTT	ACGGATTCCAAGACGTAATA
Y20	CCTATATGAGACAGGCTTGC	AACAGTCCCAAAACAATGTC
Y37	GTAATCTGGACCGTTGAGTG	AGTTATGAAAAATCGGCGTA
