# Supplementary material for: Impaired Magnesium Protoporphyrin IX Methyltransferase (ChlM) Impedes Chlorophyll Synthesis and Plant Growth in Rice
Source: Front Plant Sci. 2017 Sep 28;8:1694. doi: 10.3389/fpls.2017.01694 (PMC5626950; doi:10.3389/fpls.2017.01694)
Supplement: Supplementary file 2 [file Table2.PDF]

Table S2 Primers for detection of mutation site, confirmation of positive transgenic plants, and construction of vectors.

Primer name	Forward primer	Reverse primer
YGL18 Mutation	CGCGCCTACTTCAACTCCA	GATCATCTGCTTCGCCTCCT
*YGL18*C1	***cagtCACCTGCaaaataga***atcaggataagattaggaaa	***cagtCACCTGCaaaa***tctggggatagggttttttc
*YGL18*C2	***cagtCACCTGCaaaacaga***atcccaactccaacacccgc	***cagtCACCTGCaaaacgac***tttggtagaaggaatagggg
HYG	TCTACACAGCCATCGGTCCAG	GAAAAGTTCGACAGCGTCTCC
YGL18-YFP	*cagtGAAGACaacaac*atggcgcgcgccgccgtctc	*cagtGAAGACaataca*ctgcgaggcggcggcgatgg
GST-YGL18/*ygl18*	***cgGGATCC***ATGGCGCGCGCCGCCGTCTCCAC	***cgGAATTC***CTACTGCGAGGCGGCGGCGATGGGGA

Note: In primers of *YGL18*C1 and *YGL18*C2, underlined sequences for are used for digesting-link reaction, and the upper letters are the *Aar* I recognizing site. In primers of *YGL18-YFP*, underlined sequences for are used for digesting-link reaction, and the upper letters are the *Bbs* I recognizing site. In primers of *GST-YG1L8*/*ygl18*, the restriction enzyme sites *Bam*H I and *Eco*R I were introduced in the forward and reverse primer, respectively.
