# Supplementary material for: Impaired Magnesium Protoporphyrin IX Methyltransferase (ChlM) Impedes Chlorophyll Synthesis and Plant Growth in Rice
Source: Front Plant Sci. 2017 Sep 28;8:1694. doi: 10.3389/fpls.2017.01694 (PMC5626950; doi:10.3389/fpls.2017.01694)
Supplement: Supplementary file 3 [file Table3.PDF]

**Table S3** All primers used for qRT-PCR analysis

| Primer name | Forward primer | Reverse primer | Amplicon Length (bp) | Annealing (℃) | PCR efficiency(%) | $R^2$ |
|---|---|---|---|---|---|---|
| *ChlM*(*YGL18*) | GCTTCATCTCCACGCAGTTCT | ACATTACCTACATCGACGCAAAA | 138 | 60 | 91.0 | 0.996 |
| *Actin1* | GGAAGTACAGTGTCTGGATTGGAG | TCTTGGCTTAGCATTCTTGGGT | 155 | 60 | 98.1 | 0.995 |
| *EF-1α* | GCTGCTGCAACAAGATGGATG | CAGAGATGGGAACGAAGGGAA | 135 | 60 | 90.8 | 0.999 |
| *UBC* | GTGCAGCGAGAAAAGTCAGC | GAACTTGCGGAGGAAGGAGAG | 172 | 60 | 95.0 | 0.998 |
| *TI* | CGACATCATCAACTCCGCCAC | CCTCTTCAGACATCTTCCCACG | 83 | 60 | 97.4 | 0.997 |
| *ARF* | ATGAAAGGAAGACATGGCGG | TGGTGGTGGAACCTAAAGAGC | 126 | 60 | 95.2 | 0.999 |
| *Profilin-2* | CCAACTGGTCTTTTCCTTGGG | GGGGTCATCGGCTCATCATAG | 152 | 60 | 93.9 | 0.999 |
| *Edf* | TCCGAACCAGCAGATCATCG | GCATGGTATCAAAAGACCCAGC | 158 | 60 | 90.2 | 0.999 |
| *PtfS* | GTACACACAAAACCTAGCCACCTCT | GCCTCTTACAAACTTCAACACACG | 75 | 60 | 97.6 | 0.999 |
