# Supplementary material for: Impaired Magnesium Protoporphyrin IX Methyltransferase (ChlM) Impedes Chlorophyll Synthesis and Plant Growth in Rice
Source: Front Plant Sci. 2017 Sep 28;8:1694. doi: 10.3389/fpls.2017.01694 (PMC5626950; doi:10.3389/fpls.2017.01694)
Supplement: Supplementary file 4 [file Table4.PDF]

Table S4 Segregation of F_2 populations from four crosses

Cross	Green plants	Yellow-green plants	Total	χ^2(3:1)	*P* value
Nipponbare/*ygl18*	101	38	139	0.29	0.86
ygl18/ Nipponbare	383	130	513	0.02	0.99
02428/*ygl18*	336	103	439	0.47	0.79
ygl18/9311	992	340	1332	0.17	0.92

Note： The female parent/the male parent are showed in the cross. Green leaf and yellow-green leaf phenotypes were determined by visual inspection. $\chi^2 < \chi^2_{0.05} = 3.84$ is for 3:1 segregation ratio. *P*>0.05 is considered as significant.
