# Supplementary material for: Impaired Magnesium Protoporphyrin IX Methyltransferase (ChlM) Impedes Chlorophyll Synthesis and Plant Growth in Rice
Source: Front Plant Sci. 2017 Sep 28;8:1694. doi: 10.3389/fpls.2017.01694 (PMC5626950; doi:10.3389/fpls.2017.01694)
Supplement: Supplementary file 5 [file Table5.PDF]

Table S5 Base sequencing of the mutation site in different rice cultivars and F_2 plants

Rice cultivars		F_2 green plants		F_2 yellow-green plants	
ygl18	T	G1	C	Y1	T
Guangzhan63S	C	G2	C/T	Y2	T
9311	C	G3	C	Y3	T
02428	C	G4	C	Y4	T
Nipponbare	C	G5	C/T	Y5	T
ZH11	C	G6	C/T	Y6	T
Kongyu131	C	G7	C/T	Y7	T
YTB	C	G8	C	Y8	T
Chuanxiang29B	C	G9	C	Y9	T
Songjin13	C	G10	C	Y10	T

Note: F_2 green plants (G1-G10) and yellow-green plants (Y1-Y10) were randomly selected from F_2 population of *ygl18* and 9311.
