# Supplementary material for: Impaired Magnesium Protoporphyrin IX Methyltransferase (ChlM) Impedes Chlorophyll Synthesis and Plant Growth in Rice
Source: Front Plant Sci. 2017 Sep 28;8:1694. doi: 10.3389/fpls.2017.01694 (PMC5626950; doi:10.3389/fpls.2017.01694)
Supplement: Supplementary file 6 [file Image1.PDF]

Fig. S1

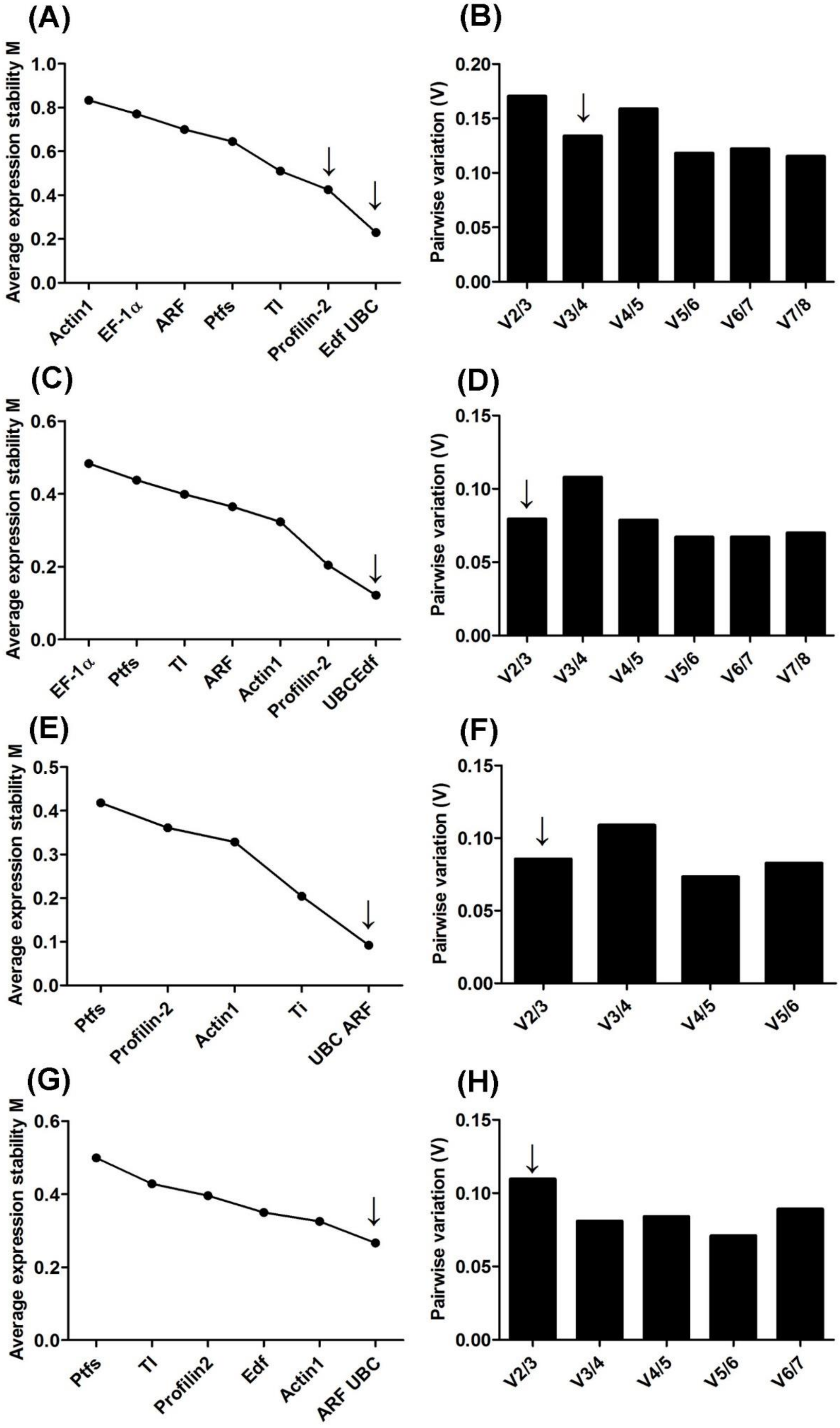

(A)
Average expression stability M
1.0
0.8
0.6
0.4
0.2
0.0
Actin1
EF-1α
ARF
Ptfs
TI
Profilin-2
Edf UBC
(B)
Pairwise variation (V)
0.20
0.15
0.10
0.05
0.00
V2/3
V3/4
V4/5
V5/6
V6/7
V7/8
(C)
Average expression stability M
0.6
0.4
0.2
0.0
EF-1α
Ptfs
TI
ARF
Actin1
Profilin-2
UBCEdf
(D)
Pairwise variation (V)
0.15
0.10
0.05
0.00
V2/3
V3/4
V4/5
V5/6
V6/7
V7/8
(E)
Average expression stability M
0.5
0.4
0.3
0.2
0.1
0.0
Ptfs
Profilin-2
Actin1
Ti
UBC ARF
(F)
Pairwise variation (V)
0.15
0.10
0.05
0.00
V2/3
V3/4
V4/5
V5/6
(G)
Average expression stability M
0.6
0.4
0.2
0.0
Ptfs
Tl
Profilin2
Edf
Actin1
ARF UBC
(H)
Pairwise variation (V)
0.15
0.10
0.05
0.00
V2/3
V3/4
V4/5
V5/6
V6/7

Fig. S1 Determination of reference genes for normalization in qRT-PCR analysis. (A, C, E, G) Ranking of reference genes for recommending multiple reference genes by geNorm to normalize gene expression in specific experimental conditions. *Arrow* indicated the best combination of multiple reference genes for normalization. (B, D, F, H) Determination of the optimal number of reference genes for accurate normalization by pairwise variation (V). *Arrow* indicated the optimal number of reference genes for normalization. (A-B) Combination of *Edf*, *UBC* and *Profilin-2* as reference genes for samples of root, leaf, stem and panicle. (C-D) Combination of *UBC* and *Edf* as reference genes for leaf samples of 15, 45, 90 and 130 day-old plants. (E-F) Combination of *UBC* and *ARF* as reference genes for the first, second and third leaf samples. (G-H) Combination of *UBC* and *ARF* as reference genes for daily expression patterns under the short-day and long-day conditions.
