# Supplementary material for: Impaired Magnesium Protoporphyrin IX Methyltransferase (ChlM) Impedes Chlorophyll Synthesis and Plant Growth in Rice
Source: Front Plant Sci. 2017 Sep 28;8:1694. doi: 10.3389/fpls.2017.01694 (PMC5626950; doi:10.3389/fpls.2017.01694)
Supplement: Supplementary file 8 [file Image3.PDF]

Fig. S3

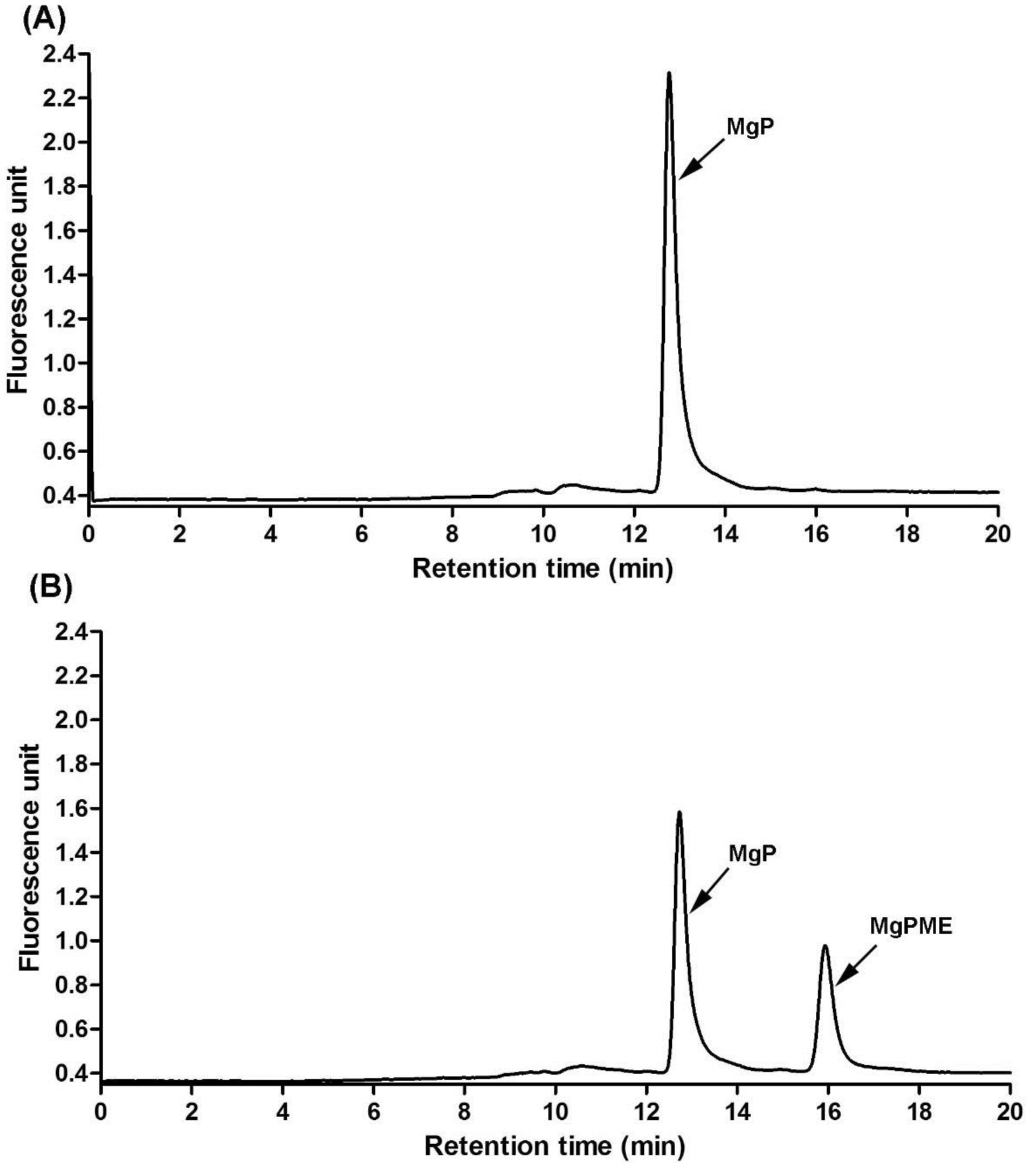

Fig. S3 Standard curves with the authentic standards when identifying MgP and MgPME in leaf samples. (A) Position of MgP. (B) Position of chemically synthesized MgPME by using MgP. The arrows indicate the peak positions of the substrate MgP (about 12.7 min) and the product MgPME (about 15.9 min).
