# Supplementary material for: Impaired Magnesium Protoporphyrin IX Methyltransferase (ChlM) Impedes Chlorophyll Synthesis and Plant Growth in Rice
Source: Front Plant Sci. 2017 Sep 28;8:1694. doi: 10.3389/fpls.2017.01694 (PMC5626950; doi:10.3389/fpls.2017.01694)
Supplement: Supplementary file 9 [file Image4.PDF]

Fig. S4

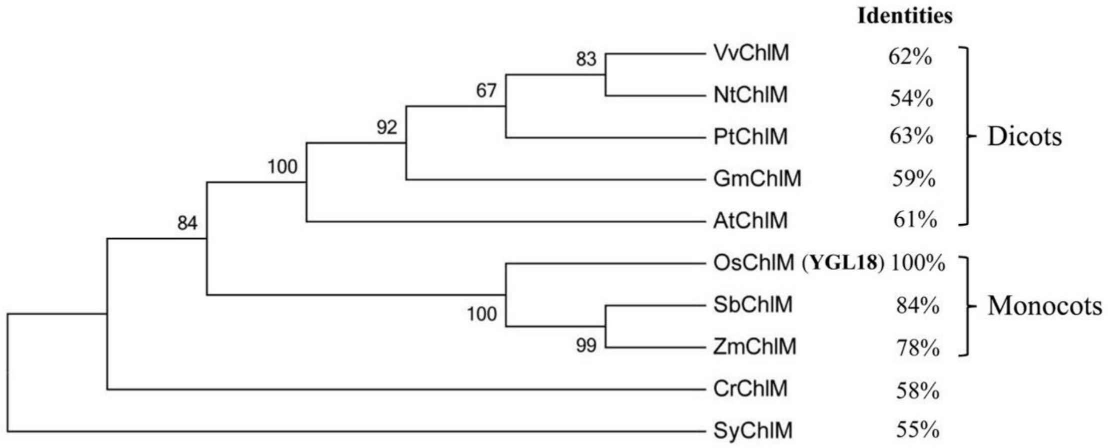

Fig. S4 Bioinformatic analysis of ChlM homologues in different photosynthetic organisms. The phylogenetic tree was constructed based on the full-length amino acid sequences of ChlMs. Sequence identities with YGL18 protein are indicated for different ChlMs analyzed by NCBI blastp. The protein sequences are introduced the same as in Fig. 5.
