# Supplementary material for: Impaired Magnesium Protoporphyrin IX Methyltransferase (ChlM) Impedes Chlorophyll Synthesis and Plant Growth in Rice
Source: Front Plant Sci. 2017 Sep 28;8:1694. doi: 10.3389/fpls.2017.01694 (PMC5626950; doi:10.3389/fpls.2017.01694)
Supplement: Supplementary file 10 [file Image5.PDF]

Fig. S5

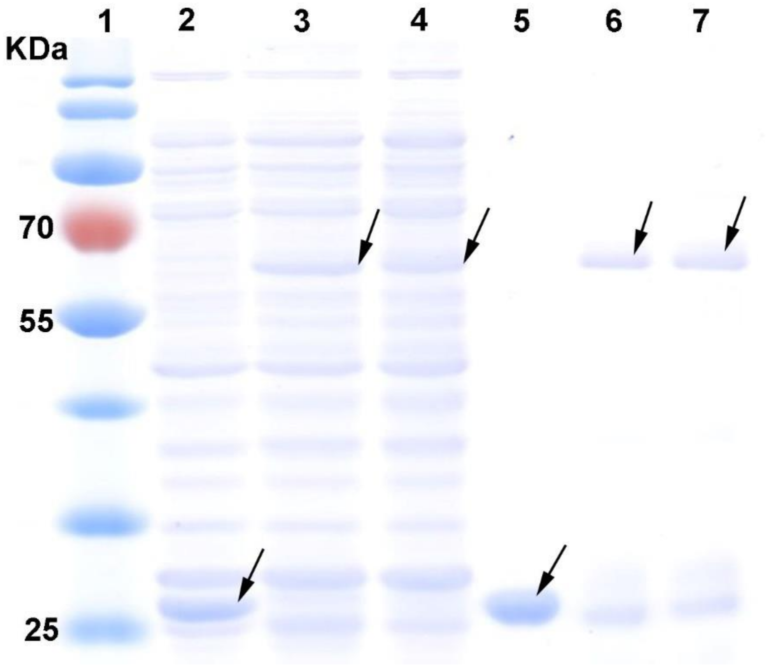

Fig. S5 SDS/PAGE of in-vitro expressed proteins. Lane 1, prestained protein ladder. Total soluble proteins from *E.coli* cells expressing control empty vector (lane 2), recombinant YGL18 (lane 3) and recombinant ygl18 (lane 4). Purified GST (lane 5), GST-YGL18 (lane 6) and GST-ygl18 (lane 7). Bands of GST, GST-YGL18 and GST-ygl18 are indicated by arrows, respectively.
