# Supplementary material for: Impaired Magnesium Protoporphyrin IX Methyltransferase (ChlM) Impedes Chlorophyll Synthesis and Plant Growth in Rice
Source: Front Plant Sci. 2017 Sep 28;8:1694. doi: 10.3389/fpls.2017.01694 (PMC5626950; doi:10.3389/fpls.2017.01694)
Supplement: Supplementary file 11 [file Image6.PDF]

Fig. S6

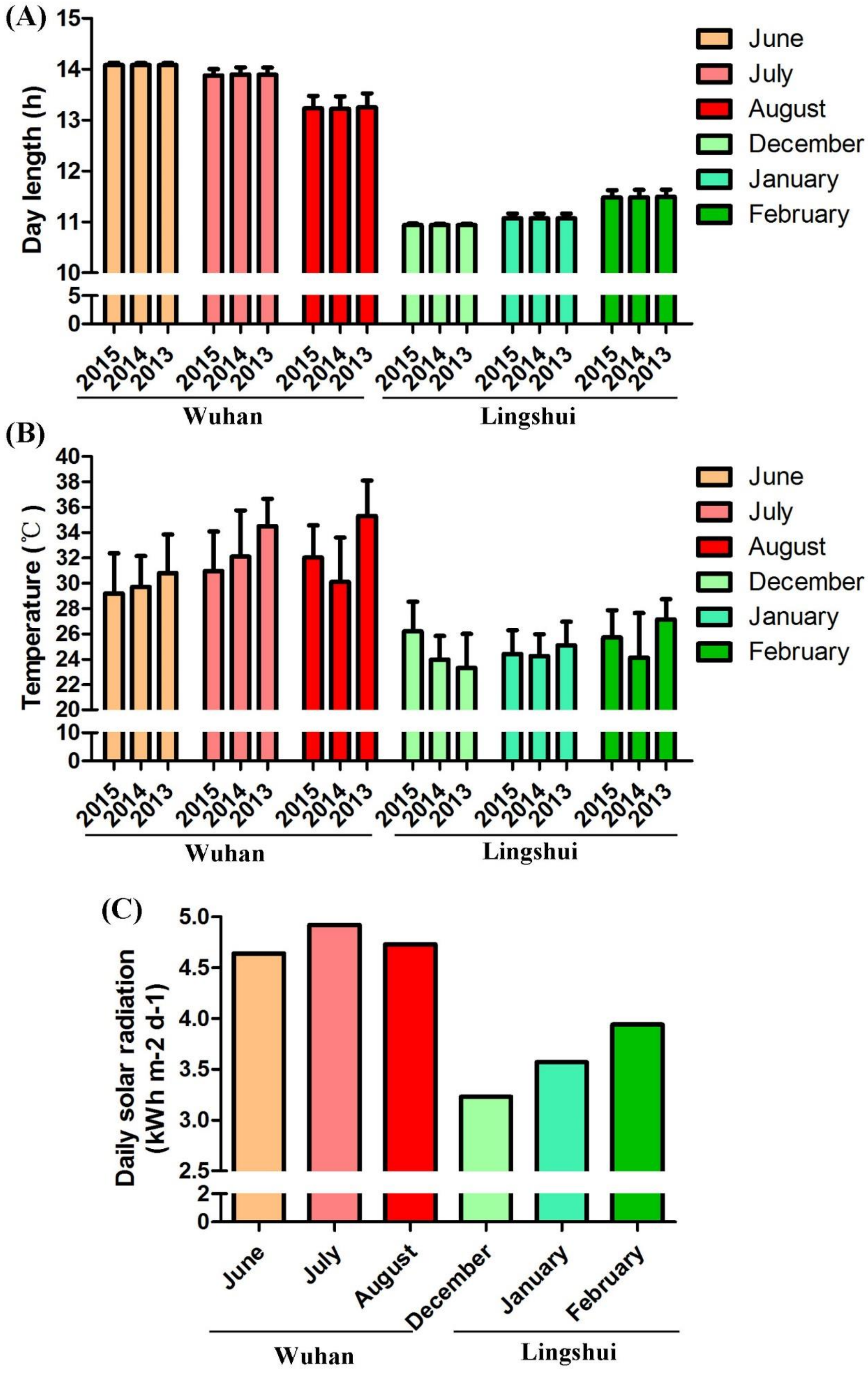

Fig. S6 Condition differences of the day length, temperature and solar radiation between Wuhan

and Lingshui in the planting period. (A) Monthly averaged day length in Wuhan (June, July and August) and Lingshui (December, January and February). Data are selected from years 2015, 2014 and 2013 on the website (http://richurimo.51240.com/zhongguo__richurimo/). (B) Monthly averaged temperature in Wuhan (June, July and August) and Lingshui (December, January and February). Data are selected from years 2015, 2014 and 2013 on the website (http://lishi.tianqi.com/). (C) Daily solar radiation in Wuhan (June, July and August) and Lingshui (December, January and February). Data are monthly averaged from 22 years by Atmospheric Science Data Center (https://eosweb.larc.nasa.gov/cgi-bin/sse/retscreen.cgi?&email=rets@nrcan.gc.ca&step=1&p=&lat=2931&submit=Submit&lon=113115).
